# Utility of ctDNA in predicting response to neoadjuvant chemoradiotherapy and prognosis assessment in locally advanced rectal cancer: A prospective cohort study

**DOI:** 10.1371/journal.pmed.1003741

**Published:** 2021-08-31

**Authors:** Yaqi Wang, Lifeng Yang, Hua Bao, Xiaojun Fan, Fan Xia, Juefeng Wan, Lijun Shen, Yun Guan, Hairong Bao, Xue Wu, Yang Xu, Yang Shao, Yiqun Sun, Tong Tong, Xinxiang Li, Ye Xu, Sanjun Cai, Ji Zhu, Zhen Zhang

**Affiliations:** 1 Department of Radiation Oncology, Fudan University Shanghai Cancer Center, Shanghai, China; 2 Department of Oncology, Shanghai Medical College, Fudan University, Shanghai, China; 3 Shanghai Key Laboratory of Radiation Oncology, Shanghai, China; 4 Translational Medicine Research Institute, Geneseeq Technology Inc., Toronto, Canada; 5 Cyberknife Center, Department of Neurosurgery, Huashan Hospital, Fudan University, Shanghai, China; 6 Nanjing Geneseeq Technology Inc., Nanjing, China; 7 School of Public Health, Nanjing Medical University, Nanjing, China; 8 Department of Radiology, Fudan University Shanghai Cancer Center, Shanghai, China; 9 Department of Colorectal Surgery, Fudan University Shanghai Cancer Center, Shanghai, China; 10 Cancer Hospital of the University of Chinese Academy of Sciences (Zhejiang Cancer Hospital), Hangzhou, China; 11 Institute of Cancer and Basic Medicine (IBMC), Chinese Academy of Sciences, Hangzhou, China; 12 Zhejiang Key Laboratory of Radiation Oncology, Hangzhou, China; Peter MacCallum Cancer Centre, AUSTRALIA

## Abstract

**Background:**

For locally advanced rectal cancer (LARC) patients who receive neoadjuvant chemoradiotherapy (nCRT), there are no reliable indicators to accurately predict pathological complete response (pCR) before surgery. For patients with clinical complete response (cCR), a “Watch and Wait” (W&W) approach can be adopted to improve quality of life. However, W&W approach may increase the recurrence risk in patients who are judged to be cCR but have minimal residual disease (MRD). Magnetic resonance imaging (MRI) is a major tool to evaluate response to nCRT; however, its ability to predict pCR needs to be improved. In this prospective cohort study, we explored the value of circulating tumor DNA (ctDNA) in combination with MRI in the prediction of pCR before surgery and investigated the utility of ctDNA in risk stratification and prognostic prediction for patients undergoing nCRT and total mesorectal excision (TME).

**Methods and findings:**

We recruited 119 Chinese LARC patients (cT3-4/N0-2/M0; median age of 57; 85 males) who were treated with nCRT plus TME at Fudan University Shanghai Cancer Center (China) from February 7, 2016 to October 31, 2017. Plasma samples at baseline, during nCRT, and after surgery were collected. A total of 531 plasma samples were collected and subjected to deep targeted panel sequencing of 422 cancer-related genes. The association among ctDNA status, treatment response, and prognosis was analyzed. The performance of ctDNA alone, MRI alone, and combining ctDNA with MRI was evaluated for their ability to predict pCR/non-pCR.

Ranging from complete tumor regression (pathological tumor regression grade 0; pTRG0) to poor regression (pTRG3), the ctDNA clearance rate during nCRT showed a significant decreasing trend (95.7%, 77.8%, 71.1%, and 66.7% in pTRG 0, 1, 2, and 3 groups, respectively, *P =* 0.008), while the detection rate of acquired mutations in ctDNA showed an increasing trend (3.8%, 8.3%, 19.2%, and 23.1% in pTRG 0, 1, 2, and 3 groups, respectively, *P* = 0.02). Univariable logistic regression showed that ctDNA clearance was associated with a low probability of non-pCR (odds ratio = 0.11, 95% confidence interval [95% CI] = 0.01 to 0.6, *P* = 0.04). A risk score predictive model, which incorporated both ctDNA (i.e., features of baseline ctDNA, ctDNA clearance, and acquired mutation status) and MRI tumor regression grade (mrTRG), was developed and demonstrated improved performance in predicting pCR/non-pCR (area under the curve [AUC] = 0.886, 95% CI = 0.810 to 0.962) compared with models derived from only ctDNA (AUC = 0.818, 95% CI = 0.725 to 0.912) or only mrTRG (AUC = 0.729, 95% CI = 0.641 to 0.816). The detection of potential colorectal cancer (CRC) driver genes in ctDNA after nCRT indicated a significantly worse recurrence-free survival (RFS) (hazard ratio [HR] = 9.29, 95% CI = 3.74 to 23.10, *P* < 0.001). Patients with detectable driver mutations and positive high-risk feature (HR_feature) after surgery had the highest recurrence risk (HR = 90.29, 95% CI = 17.01 to 479.26, *P* < 0.001). Limitations include relatively small sample size, lack of independent external validation, no serial ctDNA testing after surgery, and a relatively short follow-up period.

**Conclusions:**

The model combining ctDNA and MRI improved the predictive performance compared with the models derived from individual information, and combining ctDNA with HR_feature can stratify patients with a high risk of recurrence. Therefore, ctDNA can supplement MRI to better predict nCRT response, and it could potentially help patient selection for nonoperative management and guide the treatment strategy for those with different recurrence risks.

## Abstract 2

Zhen Zhang and colleagues conducted a cohort study to investigate the utility of circulating tumor DNA in predicting response to neoadjuvant chemoradiotherapy in patients with locally advanced rectal cancer in China.

## Author summary

**Why was this study done?:**

- For patients with locally advanced rectal cancer (LARC) who achieve clinical complete response (cCR) after neoadjuvant chemoradiotherapy (nCRT), a “Watch and Wait” (W&W) strategy can be used to improve the quality of life of these patients.
- cCR does not necessarily mean pathological complete response (pCR) or the absence of minimal residual disease (MRD). Patients with MRD who receive W&W approach may have a high risk for local recurrence and distance metastasis.
- There are no reliable biomarkers for the prediction of pCR/non-pCR currently.
- Circulating tumor DNA (ctDNA) is a cell-free DNA derived from tumor cells and has been proven to be a sensitive biomarker for tumor burden.
- ctDNA testing alone or combining with magnetic resonance imaging (MRI) may improve the prediction of pCR/non-pCR, thereby guiding patient selection for “W&W” approach.
- ctDNA may also be used for the postoperative risk stratification in LARC patients receiving nCRT and surgery.

**What did the researchers do and find?:**

- We conducted a prospective cohort study including 119 LARC patients undergoing nCRT followed by total mesorectal excision (TME). We collected 531 serial plasma samples at baseline, during nCRT, and after surgery and performed next-generation sequencing using a panel containing 422 cancer-related genes.
- We found that baseline ctDNA features, as well as the clearance of ctDNA during nCRT, were significantly correlated with pCR status.
- By establishing predictive models based on ctDNA alone, MRI alone, and combination of ctDNA and MRI, we found that the performance of the combining model in predicting pCR/non-pCR was significantly better than ctDNA alone or MRI alone.
- We also demonstrated that ctDNA testing combining with high-risk pathological features could achieve better risk stratification for postoperative recurrence.

**What do these findings mean?:**

- Combining ctDNA testing with MRI can improve the prediction of pCR/non-pCR after nCRT, identify patients with MRD, and help patient selection for W&W strategy.
- ctDNA can be used in recurrence risk assessment and stratification in patients receiving nCRT and surgery.
- The findings from this study should be validated in larger studies. Moreover, whether utility of ctDNA in patient selection for W&W strategy can reduce local regrowth (LR) or distant metastasis (DM) in the patients also needs clinical trial to confirm.

## Introduction

Colorectal cancer (CRC) is the third most common cancer and the fourth leading cause of cancer-related mortality worldwide [1], and locally advanced rectal cancer (LARC) constitutes up to 15% of all CRC cases [2]. Currently, neoadjuvant chemoradiotherapy (nCRT) followed by total mesorectal excision (TME) and adjuvant chemotherapy is the standard treatment for LARC recommended by the National Comprehensive Cancer Network (NCCN) guidelines [3]. Around 10% to 35% of patients treated with nCRT can achieve pathological complete response (pCR) [4], and these pCR patients would have less local recurrence, less distant metastasis (DM), and improved 5-year overall survival [5,6]. As pCR cannot be determined prior to surgery, clinical complete response (cCR), defined as undetectable tumor signs after nCRT by clinical examinations including magnetic resonance imaging (MRI), endoscopy, and digital rectal examination (DRE), has been used as a surrogate of pCR to guide the selection of patients appropriate for “Watch and Wait” (W&W) strategy [7,8]. However, the concordance between cCR and pCR is suboptimal [9], and cCR-based patient selection may not be sufficient to guide W&W, which results in worse clinical outcomes than the standard surgical treatment in some patients [10]. Therefore, accurately predicting patients’ response to nCRT before surgery will help direct treatment strategies.

Circulating tumor DNA (ctDNA) has been used as an indicator of treatment response to chemotherapy in metastatic CRC [11,12], and ctDNA also showed promising results in early detection of recurrence in colon cancer patients receiving adjuvant chemotherapy [13]. However, not until recently has the role of ctDNA in evaluating tumor response to nCRT and prognosis in LARC patients been investigated [14–17]. The potential role of ctDNA to guide nCRT treatment in LARC patients has not been fully evaluated. In the present study, we investigated the potential of using ctDNA monitoring in combination with MRI information to predict pCR/non-pCR status and explored the prognostic value of ctDNA for LARC patients undergoing nCRT treatment.

## Materials and methods

### Study design

The study was a prospective cohort study and was approved by the Human Research Ethics Committee of Fudan University Shanghai Cancer Center. All patients provided written informed consent. From February 7, 2016 to October 31, 2017, a total of 119 LARC (cT3-4/N0-2, M0) patients were enrolled, and they received nCRT (50Gy/25 fractions; concurrent capecitabine + irinotecan chemotherapy) and 1 cycle of interval chemotherapy (CAPIRI, capecitabine + irinotecan), followed by TME and 5 cycles of adjuvant chemotherapy (CAPOX, capecitabine + oxaliplatin). Detailed information about the regimen was in S1 Text. This regimen was motivated by a Phase III randomized controlled clinical trial (CinClare, NCT02605265) conducted in our cancer center, which proved that the addition of irinotecan in nCRT could achieve a significantly higher pCR rate of 30%, compared with a pCR rate of 15% in the capecitabine arm. The toxicities were acceptable and under control [18].

**Fig 1:**
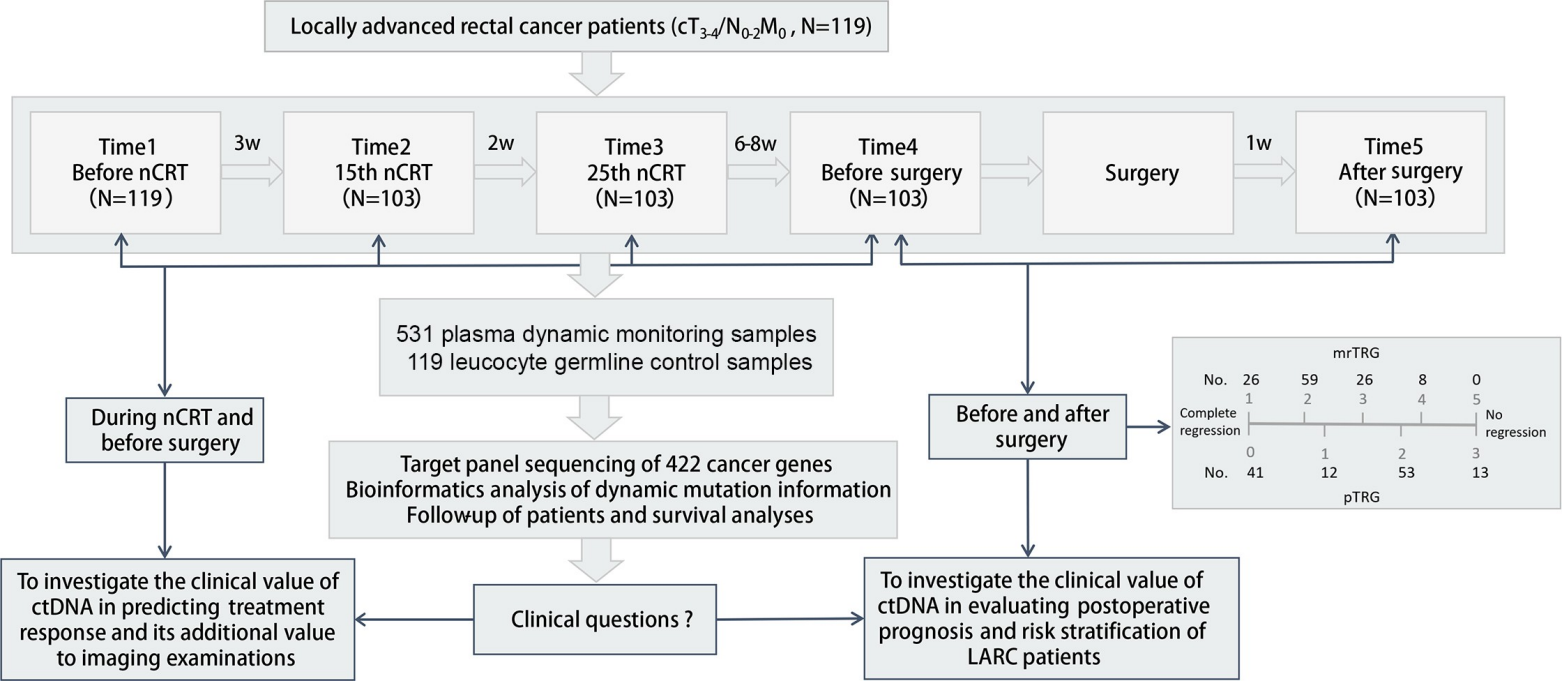
Study design, sample collection, study objectives, and work scheme of the present study. ctDNA, circulating tumor DNA; LARC, locally advanced rectal cancer; mrTRG, magnetic resonance tumor regression grade; nCRT, neoadjuvant chemoradiotherapy; pTRG, pathological tumor regression grade.

Plasma samples were collected at the following time points: before nCRT (Time1), at the 15th (Time2) and the 25th (Time3) fractions of nCRT, 0 to 1 days before surgery (Time4), and 5 to 12 days after surgery (Time5). Baseline plasma samples were collected for all 119 patients, while 16 patients failed to fulfill the subsequent 4 time points of sample collection, leaving 103 patients who completed the whole course of the study. Pathological tumor regression grade (pTRG) was evaluated according to the 8th American Joint Committee on Cancer (AJCC) Staging Manual [19]. pCR was defined as pathological T0N0M0 and pTRG = 0. The pTRG and pCR statuses were evaluated by 2 independent pathologists. If their conclusions were inconsistent, it was evaluated again by a third pathologist. Pathology reviewers were blinded to MRI results. MRI assessment of TRG (mrTRG) was evaluated according to the MERCURY experience [20]. Baseline and pre-surgery MRI images were compared with the reviews of 2 independent radiologists. It was sent to a third radiologist if there were inconsistencies. MRI reviewers were blinded to pathological results. Generally, definition of cCR needs data from MRI, endoscopy, and DRE. In this study, due to lack of endoscopy and DRE information, for the convenience of comparing pCR, cCR was defined as mrTRG1 representing the complete response in MRI. The criteria for judging pTRG and mrTRG were shown in Table 1. Detailed information on study design, patient enrollment, sample collection, study flow, and patient number with various pTRG and mrTRG were shown in Fig 1 and S1 Text.

**Table 1:** Criteria for classifying pTRG and mrTRG.

	Grade	Definition
**mrTRG**	1	Complete regression (absence of tumor signal and barely visible treatment related scar)
	2	Good regression (predominant low signal intensity fibrosis with no obvious areas of intermediate signal intensity)
	3	Moderate regression (low signal intensity fibrosis predominates but there are obvious areas of intermediate signal intensity)
	4	Slight regression (little areas of low signal intensity fibrosis or mucin but mostly tumor)
	5	No regression (intermediate signal intensity, same appearances as original tumor)
**pTRG**	0	No residual tumor cells
	1	Single cell or small group of cells
	2	Residual cancer with desmoplastic response
	3	Minimal evidence of tumor response

mrTRG, magnetic resonance tumor regression grade; pTRG, pathological tumor regression grade.

### ctDNA sequencing and bioinformatics analysis

A total of 531 dynamic plasma samples and 119 leukocyte germline control samples were collected and subjected to panel sequencing of 422 cancer-related genes. The 422-gene panel includes genes associated with targeted medicines approved by Food and Drug Administration (FDA) or recommended by the NCCN guideline, genes involved in the major signaling pathways regulating cancer cell survival and proliferation, and potential cancer driver genes; it covers CRC-related genes, such as those associated with CRC development or prognosis (*APC*, *TP53*, *KRAS*, *BRAF*, *PTEN*, *PIK3CA*, etc.) and hereditary CRC (*MLH1*, *MLH3*, *MSH2*, *MSH6*, etc.) (S1 Table). The panel has been used in the study of lung cancer immunotherapy [21], genomic profiling of melanoma [22], and drug resistance of pancreatic cancer [23]. The average sequencing depth was approximately 4,000X. Baseline ctDNA sequencing and analysis were applied to all 119 patients. A total of 103 patients completed the whole study (Fig 1), 89 of whom had detectable ctDNA mutations at baseline. Detailed information about sample preparation, sequencing, data processing, and bioinformatics analysis was provided in S1 Text. We tracked the dynamic change of the mutation with the highest variant allele frequency (VAF) at baseline in each patient. ctDNA clearance meant unable to detect the mutation with highest VAF at certain time points, and T234_clearance meant unable to detect the mutation in all 3 preoperative time points (Time 2, 3, and 4 persistent clearance). To reduce potential false positives, genetic alterations that were detected in at least 2 time points after baseline were used for acquired mutations analysis. Detection of colorectal driver gene mutations meant detection of mutations in any 1 of 15 potential colorectal driver genes, which were introduced by Tie and colleagues [24] (S2 Table), at certain time points (e.g., T4 and T5).

### Statistical analysis

Analyses were performed according to a prespecified analysis plan (S1 Analysis Plan). Baseline feature analyses including the distribution of patients with baseline genomic or clinical features in pCR/non-pCR or pTRG groups and the association of baseline features with either pCR status or patients’ prognosis were performed on all 119 patients. The analyses of acquired mutation and the association between the detection of driver gene mutations and prognosis were performed on 103 patients who had serial ctDNA test data (i.e., completed the whole study). Analyses involving ctDNA clearance, such as the association of ctDNA clearance with pCR status and pCR predictive model construction, were performed on 89 patients who had both detectable baseline mutations and serial ctDNA test data.

The comparison of the proportion of patients with certain clinical or genetic features in pCR and non-pCR groups was performed by Fisher exact test (two-sided test), and their increasing or decreasing trend in different TRG groups were analyzed using Cochran–Armitage test (one-sided test). For multiple tests, the adjusted *P* value was calculated by Benjamini–Hochberg method.

We used univariable logistic regression to investigate the association of baseline ctDNA features such as detection of gene or Kyoto Encyclopedia of Genes and Genomes (KEGG) pathway mutations at baseline, as well as ctDNA dynamic change (ctDNA clearance and acquired mutation) with the probability of non-pCR. The effect of baseline pathological features, such as age, sex, and disease stage, on the probability of non-pCR was also analyzed by univariable logistic regression. We selected those features that were associated with pCR/non-pCR status to construct predictive models for pCR/non-pCR prediction. Generally, we selected features with *P* value ≤ 0.1 in the univariable logistic regression to perform multivariable logistic regression, and only features with *P* value ≤ 0.25 in the multivariable regression were kept. For KEGG pathways, we used more stringent criteria. Firstly, pathways with *P* value ≤ 0.05 in univariable logistic regression were selected; secondly, the selected pathways were included to fit a multivariable logistic regression; thirdly, pathways maintained significance (*P* value ≤ 0.05) in multivariable analysis were selected for further multivariable logistic regression including other features. We constructed 3 predictive models based on multivariable logistic regression, one contained only ctDNA information, one contained only mrTRG information, and the third one contained both ctDNA and mrTRG information. For each model, multivariable logistic regression was performed to calculate the odds ratios of each feature. In logistic regression, non-pCR was defined as the positive event, and the risk score for non-pCR of each patient was calculated based on the multivariable regression equation according to the method of Pavlou and colleagues [25] (Risk score = intercept + (b_feature 1_ × feature 1 status) + (b_feature 2_ × feature 2 status) + (b_feature 3_ × feature 3 status) +…+ (b_feature N_ × feature N status). Receiver operating characteristic (ROC) curve was plotted by “pROC” package of R [26]. Confidence interval of area under the curve (AUC) was calculated by DeLong method. Comparison of AUCs of different models was performed by DeLong method. For each model, 5-fold internal cross-validation repeating 100 times was performed to evaluate the predictive performance. Different random split of the data was used in each of the 100 repeats. At the same time, stratified cross-validation was used to ensure that the proportion of pCR cases was similar in each fold (around 25%). Data split was performed by using “splitTools” R package.

Survival analyses including Kaplan–Meier method and univariable or multivariable Cox proportional hazards model were performed using the “survival” and “survminer” R packages. All statistical analyses were performed in R-3.5.1 platform (https://www.r-project.org).

This study is reported as per the REMARK guideline (S1 REMARK Checklist).

## Results

### Patient characteristics

The median age of the 119 patients was 57 years old, with 71.4% of them being males. Most patients were in stages IIIB (66.4%) and IIIC (31.1%). Postoperative pathological examination showed that 41 (34.5%) patients were pCR. As for pTRG, 41 (34.5%), 12 (10.1%), 53 (44.5%), and 13 (10.9%) patients achieved grade 0, grade 1, grade 2, and grade 3, respectively. Univariable logistic regression showed that age and mrTRG were significantly associated with higher probability of non-pCR (S3A Table). For major clinical features except for age, their distribution in the pCR and non-pCR groups was not significantly different (S3B Table).

### Association of baseline ctDNA features and ctDNA dynamic change with the response to nCRT

Somatic mutations were detected in 100 (84.0%) patients at baseline, and detection of baseline ctDNA was not associated with treatment response or recurrence-free survival (RFS) (S1 Fig). The most commonly detected genes were *TP53*, *APC*, and *KRAS* (S2 Fig). Besides the above 3 genes, genes with relatively high mutation frequency included *KMT2B*, *NOTCH1*, and *POLD1*. Similarly, we used univariable logistic regression to investigate the association of baseline gene mutations with the probability of non-pCR, and only genes that were detected to be mutated in at least 6 patients were included. Due to the small sample size and multiple test adjustment, the adjusted *P* values of 10 included genes did not achieve statistical significance, with the detection of *APC*, *TP53*, and *POLD1* tending to be associated with a high non-pCR probability (*P* ≤ 0.1; S4A Table). We next performed univariable logistic regression analysis at the pathway level. If 1 patient had detectable mutations of any gene of a specific pathway, then the patient was considered to be mutated in that pathway. For KEGG pathways, only pathways containing at least 5 overlapping genes with the detected mutated genes in at least 8 patients of our cohort were included in the analysis. Among 125 included pathways, only 6 pathways showed significant association with pCR status (*P* ≤ 0.05, all adjusted *P* values were greater than 0.25 due to small sample size). We then included these 6 pathways into the multivariable logistic regression analysis, and only homologous recombination (HRR) and histone methyltransferase (HMT) maintained to be statistically significant (S4B Table). Subsets of HRR and HMT pathway genes that were detected in our cohort were shown in S4C Table.

We also analyzed the association between pCR status and ctDNA dynamic change, including T234_clearance and acquired mutation status. As expected, patients with T234_clearance had a lower probability of non-pCR than those with ctDNA non-clearance (odds ratio = 0.11, 95% confidence interval [95% CI] = 0.01 to 0.6, *P* = 0.04), and patients with acquired mutations during nCRT had a higher probability of non-pCR than those without acquired mutations (odds ratio = 5.56, 95% CI = 1.03 to 103.31, *P* = 0.1), although not achieving statistical significance (S4D Table).

**Fig 2:**
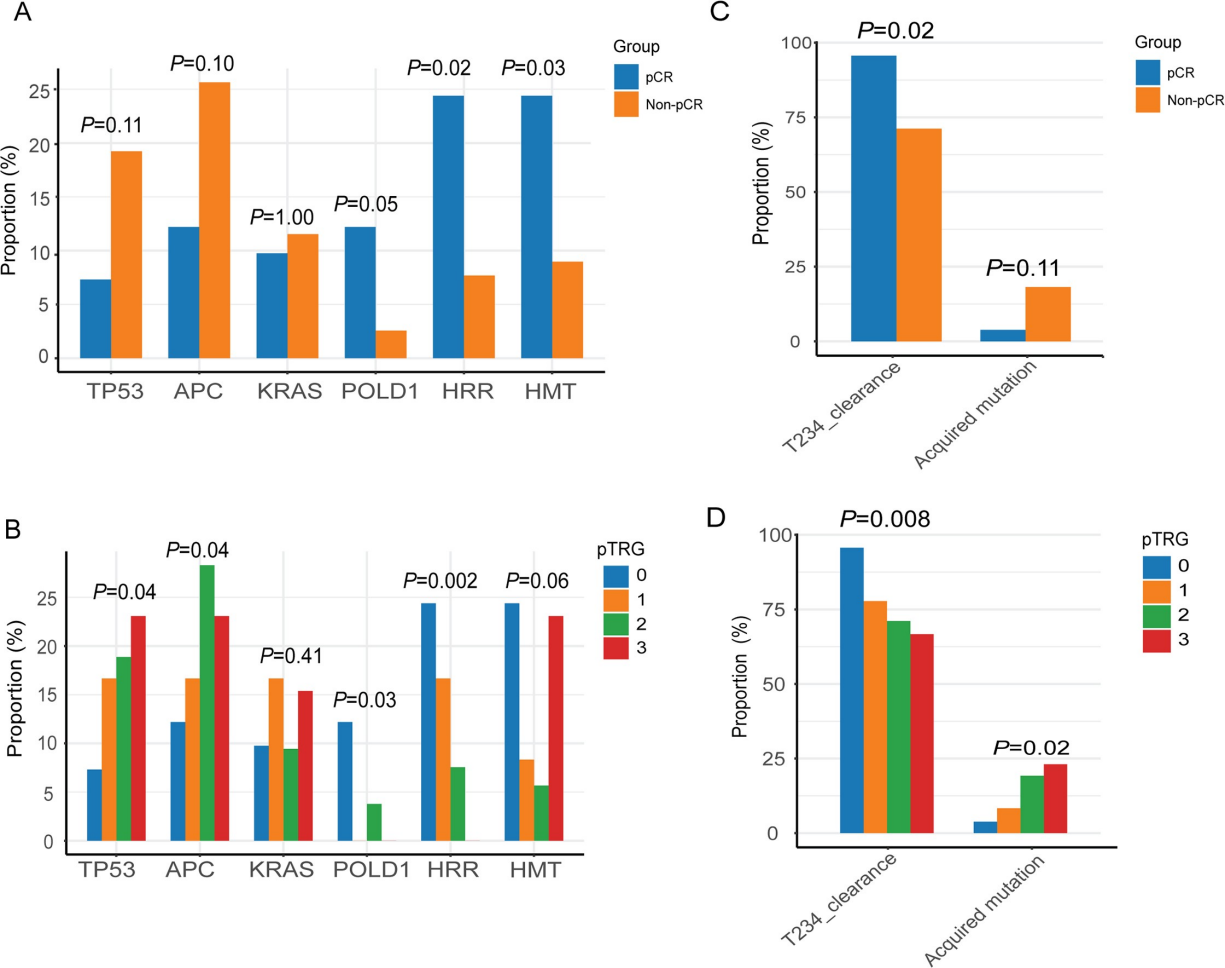
Baseline ctDNA features, ctDNA dynamic clearance, and acquisition were associated with pTRG and pCR. (A) Distribution of mutation rates of *TP53*, *APC*, *KRAS*, *POLD1*, and 2 pathways (HRR and HMT) in pCR and non-pCR groups; Fisher exact test was used to compare the difference between pCR and non-pCR groups. (B) Distribution of mutation rates of the 4 genes and 2 pathways in different TRG groups. Y axis represents the proportion of patients carrying corresponding gene mutations accounting for total patients in the corresponding TRG group. Cochran–Armitage test was used to test the increasing trend (*TP53*, *APC*, and *KRAS*) and decreasing trend (*POLD1*, HRR, and HMT) in TRG groups. (C) Proportions of patients with T234_clearance or acquired mutations in pCR and non-pCR groups. Fisher exact test was used for intergroup comparison (two-sided). (D) Proportions of patients with T234_clearance or acquired mutations in various pTRG groups. Cochran–Armitage trend test was used to test increasing trend for acquired mutation status and decreasing trend for T234_clearance from pTRG0 to pTRG3 (one-sided*)*. ctDNA, circulating tumor DNA; HMT, histone methyltransferase; HRR, homologous recombination; pCR, pathological complete response; pTRG, pathological tumor regression grade.

We next studied the distribution of patients with the above key features in various pTRG groups or pCR/non-pCR groups. We found that *TP53* and *APC* mutations were prone to be detected in patients with non-pCR and high pTRG grade (*P* = 0.11 of *TP53* and *P* = 0.10 of *APC* for pCR, *P* = 0.04 of both *TP53* and *APC* for TRG), whereas both of HRR and HMT showed an opposite trend (Fig 2A and 2B), particularly for HRR pathway (*P* = 0.002 for pTRG), suggesting that mutations in these 2 pathways may sensitize cancer cells to radiochemotherapy. Consistent with a recent study [27], *POLD1* mutations were prone to be detected in pCR group and low pTRG grade (*P* = 0.05 for pCR and *P* = 0.03 for pTRG; Fig 2A and 2B). Detection of *KRAS* mutations did not show significant different distribution among various pTRG group or pCR/non-pCR group (Fig 2A and 2B).

We then investigated T234_clearance and the acquired mutation detection rate in these LARC patients. T234_clearance rate was higher in pCR patients (*P =* 0.02; Fig 2C), and similar results were seen when grouping patients by their tumor regression grades, with T234_clearance rate showing a decreasing trend from complete regression (pTRG0) to poor regression (pTRG3) (95.7%, 77.8%, 71.1%, and 66.7% in pTRG 0, 1, 2, and 3 groups, respectively, *P =* 0.008; Figs 2D and S3A). On the other hand, the acquired mutation detection rate was higher in non-pCR patients (*P =* 0.11; Fig 2C) and showed an increasing trend from pTRG0 to pTRG3 (3.8%, 8.3%, 19.2%, and 23.1% in pTRG 0, 1, 2, and 3 groups, respectively, *P =* 0.02; Figs 2D and S3B). These results suggest that non-pCR patients not only had less clearance of baseline mutations but also acquired additional mutations during nCRT. Detailed information regarding the distribution of the patients with certain features in pTRG groups and pCR/non-pCR groups were provided in S4E Table.

### ctDNA clearance supplemented MRI to identify non-pCR patients

By using the cCR (mrTRG1) standard in our study, the pCR/non-pCR prediction accuracy for all 119 patients was 70.6% (84/119), and for 89 patients who had ctDNA clearance information, the pCR/non-pCR prediction accuracy was 73.0% (65/89) (S3A Fig). Of note, 8 out of 89 patients who were determined to be cCR by MRI were confirmed to be non-pCR after surgery (indicated by 8 arrows at the bottom of S3A Fig). Among the 8 patients, 4 of whom were T234_non-clearance (indicated by 4 blue arrows in S3A Fig), including 1 patient experienced DM 138 days after surgery. This indicates that ctDNA clearance could provide additional pCR/non-pCR prediction values for 4 out of 8 cCR-misjudged patients, while the proper diagnosis of the rest 4 patients (indicated by 4 yellow arrows in S3A Fig) might be hindered by the low tumor load after nCRT. In addition, ctDNA non-clearance seemed to be enriched in non-pCR group. Only 1 of 23 patients with pCR was ctDNA non-clearance (S3A Fig). Patients with detectable acquired mutations were also enriched in non-pCR patients (S3B Fig).

### Clearance of HRR and HMT mutations during nCRT

Among 89 patients who had detectable mutations at baseline and completed the whole sample collection and sequencing procedures, a total of 19 HRR mutations and 16 HMT mutations were detected at baseline. If clearance was defined as being cleared at all of the 3 time points before surgery (Time2, Time3, and Time4), only 1 HRR mutation [BRCA2:c.2525T>C(p.V842A)] and 1 HMT mutation [NSD1:c.2903A>G(p.K968R)] were not cleared during nCRT, representing a 5.3% non-clearance rate of HRR mutations and 6.2% non-clearance rate of HMT mutations (S4A and S4B Fig). The overall non-clearance rate was 11.9% (51 out of 429 mutations were not cleared), while HRR and HMT were among the top 5 KEGG pathways with the lowest non-clearance rate (S4C Fig), suggesting that tumor cells carrying HRR or HMT mutations may be more sensitive to nCRT, thereby, were largely cleared by nCRT. This result was in agreement with the previous finding that patients with HRR or HMT mutations were enriched in the pCR group and low pTRG group.

### Predicting pCR/non-pCR by combining ctDNA and mrTRG information

**Fig 3:**
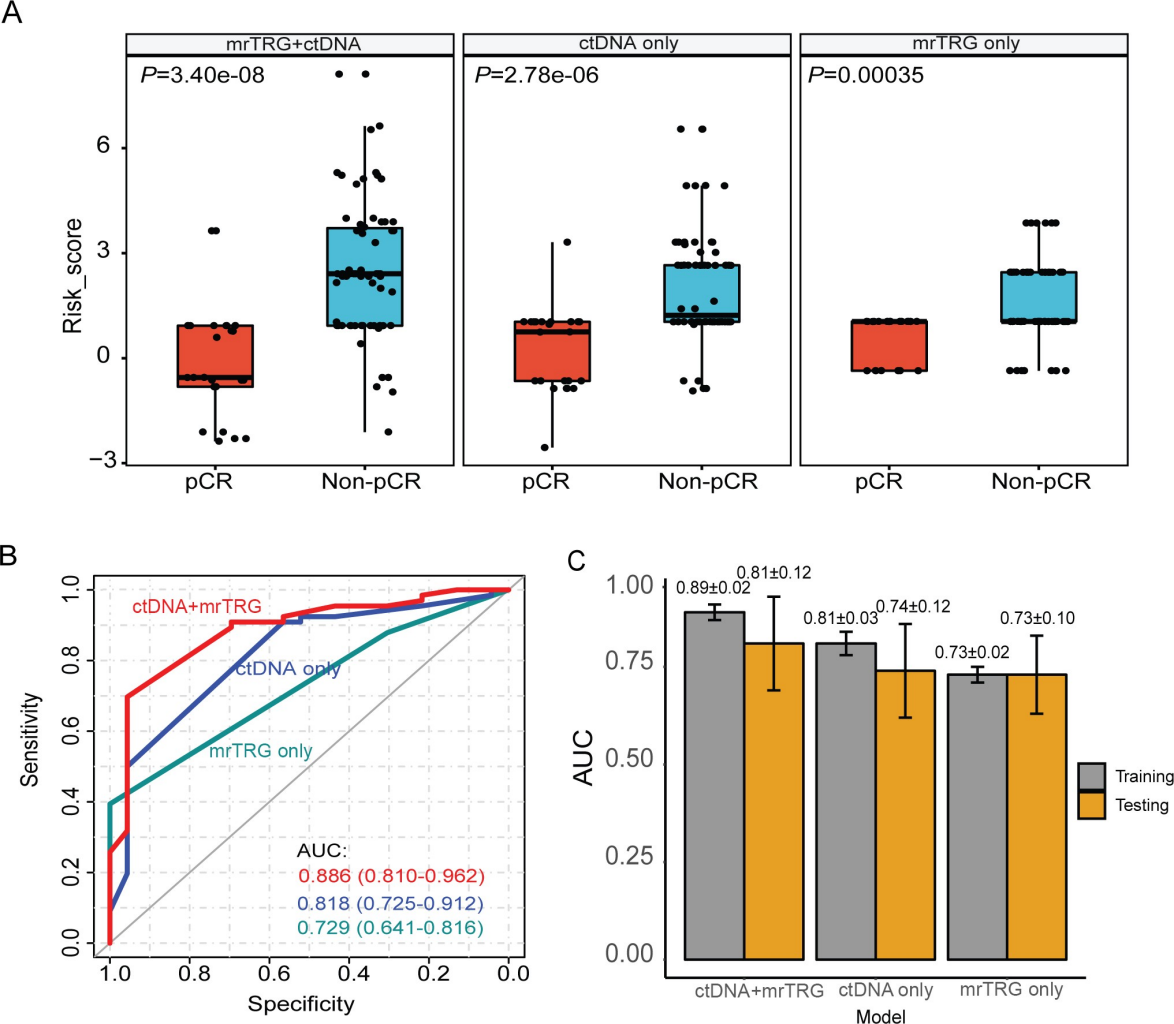
Predicting pCR/non-pCR by combining ctDNA and mrTRG information. (A) Distribution of risk scores obtained from the 3 predictive models in pCR and non-pCR groups. Wilcoxon rank sum test was used for intergroup comparison (two-sided). (B) AUC analysis of the 3 models. 95% CI of AUC was calculated by “DeLong” method. (C) Predictive performance of the 3 models was evaluated by internal 5-fold cross-validation and 100 times repeats. The numbers on the top of the bars were average AUC ± standard deviation. Construction of the 3 models refers to Materials and methods section. AUC, area under the curve; ctDNA, circulating tumor DNA; mrTRG, magnetic resonance imaging tumor regression grade; pCR, pathological complete response; 95% CI, 95% confidence interval.

Next, we investigated whether baseline genetic features and ctDNA clearance can be used to improve the prediction of patient’s pCR status. We firstly selected features with *P* value ≤ 0.1 in previous univariable logistic regression to perform multivariable logistic regression analysis. Because *POLD1* is a member of HRR pathway, only HRR mutation was selected. *APC* mutation and *TP53* mutation were largely overlapped, and the effect of *TP53* mutation on chemoradiotherapy has been confirmed by many studies; therefore, *TP53* mutation instead of *APC* mutation was selected. Features with *P* value ≤ 0.25 in multivariable regression were then selected to construct predictive models. Of note, age was not included in the models because its *P* value was greater than 0.25 in multivariable regression, although it was significant in univariable regression. Besides statistical significance, we also took account of the biological significance of these features. For example, HRR and HMT pathways have been extensively reported to be associated with radio- and chemo-sensitivity [28–30]. Lastly, in addition to the mrTRG information, 5 ctDNA features were selected, including *TP53* mutation, HRR mutation, HMT mutation, T234_clearance, and the acquired mutation status. Based on multivariable logistic regression, we constructed 3 scoring pCR/non-pCR prediction models (Statistical analysis of the Materials and methods section), which were derived from ctDNA information only, mrTRG information only, or the combination of both aspects. A total of 89 patients, consisting of 23 (25.8%) pCR patients and 66 (74.2%) non-pCR patients, who had ctDNA clearance data, were included in the model construction and evaluation. Detailed information regarding model construction and calculation of the risk score were provided in S5 Table. As shown in Fig 3A, by measuring the risk score that quantifies the chance of a patient to be non-pCR, the model incorporating both ctDNA and mrTRG (i.e., combining model) had the most significant differences between pCR and non-pCR patients (*P* = 3.40e-08, Wilcoxon test), implying its superiority in pCR/non-pCR discrimination. Consistent with this observation, the combining model had higher pCR/non-pCR prediction performance (AUC: 0.886, 95% CI: 0.810 to 0.962) than the mrTRG only model (AUC: 0.729, 95% CI: 0.641 to 0.816) (*P* < 0.001) or the ctDNA only model (AUC: 0.818, 95% CI: 0.725 to 0.912) (*P* = 0.008) (Fig 3B). Five-fold cross-validation (repeating 100 times) showed that training AUC of the combining model was 0.89 ± 0.02 and testing AUC was 0.81 ± 0.12 (Fig 3C).

### Recurrence risk assessment of LARC patients undergoing nCRT by ctDNA monitoring

**Fig 4:**
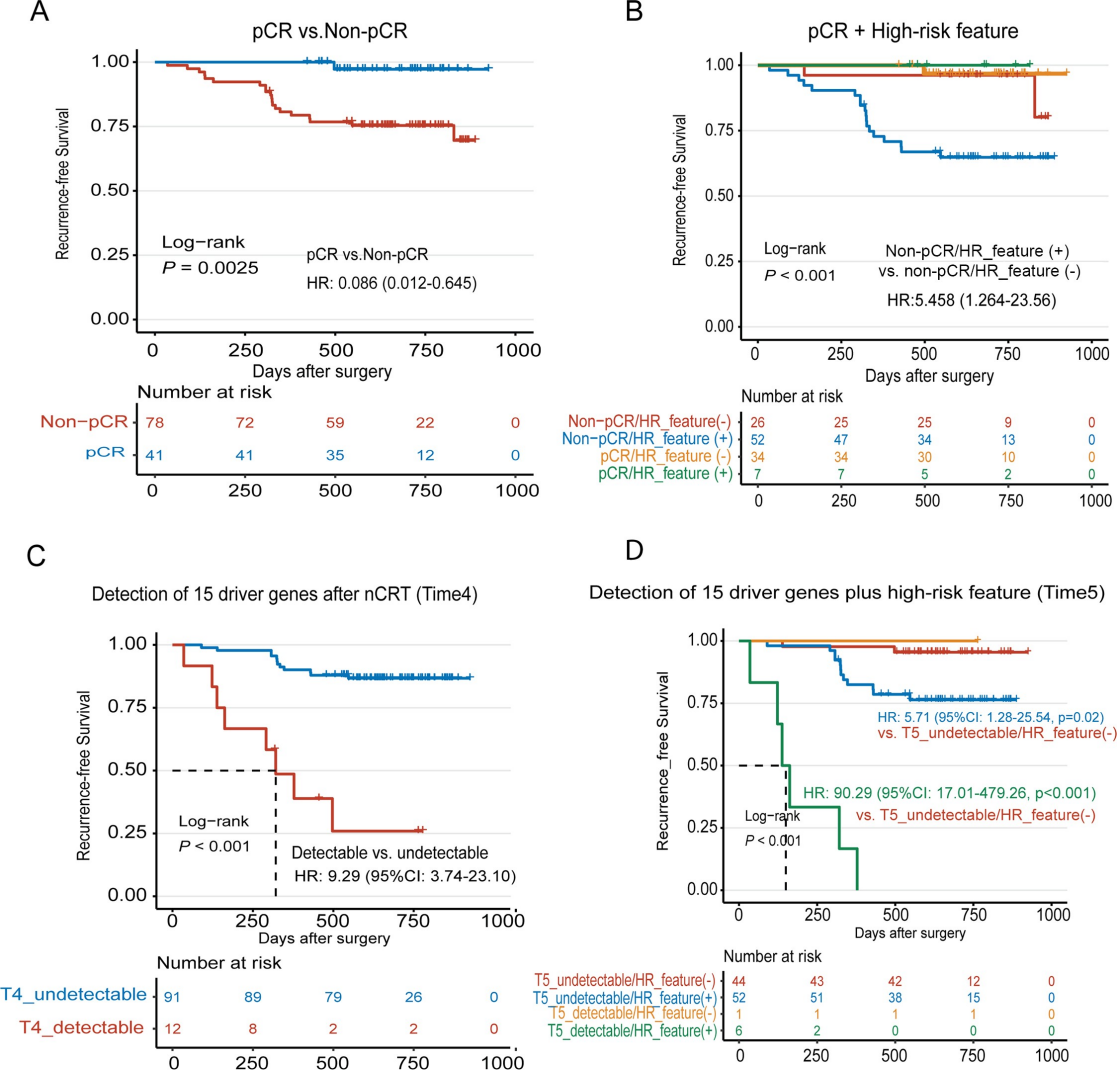
Recurrence risk assessment of LARC patients undergoing nCRT by ctDNA monitoring. (A) Kaplan–Meier analysis of RFS stratified by pCR status. All 119 patients with pCR/non-pCR information were included in the analysis. (B) Kaplan–Meier analysis of RFS stratified by pCR status plus HR_feature. HR_ features included baseline-detectable TP53 or KRAS mutation, tumor deposits, PNI, vascular invasion, and lymph node metastasis. HR_feature (+) was defined as positive in at least 1 feature, otherwise, HR_feature (−). All 119 patients with pCR/non-pCR information were included in the analysis. (C) Kaplan–Meier analysis of RFS stratified by T4_driver_gene mutation detection status (Time4, after nCRT). T4_detectable was defined as at least 1 mutation of the 15 CRC driver genes could be detected at Time4 point, otherwise, T4_undetectable. A total of 103 patients who completed the whole study were included in the analysis. (D) Kaplan–Meier analysis of RFS stratified by T5_driver_gene mutation detection status (Time5, after surgery) plus HR_feature. A total of 103 patients who completed the whole study were included in the analysis. CRC, colorectal cancer; ctDNA, circulating tumor DNA; HR, hazard ratio; HR_feature, high-risk feature; HR_feature (+), HR_feature positive; LARC, locally advanced rectal cancer; nCRT, neoadjuvant chemoradiotherapy; pCR, pathological complete response; PNI, perineural invasion; RFS, recurrence-free survival; 95% CI, 95% confidence interval.

We then studied the prognosis of these LARC patients. The median follow-up after surgery was 644 days (35 to 925 days), and 21 out of 119 (17.6%) patients progressed. As expected, pCR status was associated with RFS (*P* = 0.0025; Fig 4A), and only 1 out of 41 pCR patients relapsed during follow-up, compared with 20 relapses in 78 non-pCR patients. Of note, the detection of *TP53* or *KRAS* mutations at baseline indicated a high recurrence risk (*P* < 0.001 and *P* = 0.02, respectively; S5A and S5B Fig). Besides, several pathological features, including perineural invasion (PNI), tumor deposits, vascular invasion, and lymph node metastasis, were also associated with a high risk of recurrence (*P* = 0.014, 0.0016, 0.003 and *P* < 0.001, respectively; S5C–S5F Fig). We thereby defined a “high-risk feature (HR_feature) positive” status to indicate at least 1 of the above 6 features being positive. As a result, the majority (18/20) of non-pCR patients with recurrence were HR_feature positive (Fig 4B), suggesting that HR_feature was a key factor to determine non-pCR patient recurrence.

Lastly, we investigated the value of ctDNA monitoring for risk assessment and patient stratification. We focused on 2 time points: after nCRT (Time4) and after surgery (Time5). For ctDNA clearance, the Time4_clearance did not show significant impacts on patients’ recurrence (*P* = 0.65; S6A Fig). However, as more pathological information was available after surgery, the concurrence of ctDNA non-clearance (Time5) and HR_feature positive was found to be associated with the worst RFS (S6B Fig). Detection of potential CRC driver gene mutations after nCRT was associated with a much worse RFS (hazard ratio [HR]: 9.29; 95% CI: 3.74 to 23.10, *P* < 0.001; Fig 4C). Similarly, in combination with HR_feature, driver ctDNA detection could make a more precise risk evaluation (Fig 4D), and all 6 patients with the concurrence of detectable driver mutations and positive HR_feature relapsed. Thus, patients could be stratified into 3 risk groups. The low-risk group included double-negative patients with a 2-year RFS of 95.5%. The intermediate-risk group included patients with positive HR_feature but undetectable driver mutations; their 2-year RFS was 76.4% and HR was 5.71 (95% CI: 1.28 to 25.54; *P* = 0.02, relative to the low-risk group; Fig 4D). The high-risk group included double-positive patients; their 2-year RFS was 0 and HR was 90.29 (95% CI: 17.01 to 479.26, *P* < 0.001, relative to the low-risk group; Fig 4D). There was only 1 patient who was driver mutation detectable but HR_feature negative; therefore, the risk of this group could not be evaluated. Multivariable Cox analysis showed that HR_feature and Time5 driver mutation detection were independent risk factors for postoperative recurrence (S6 Table). Overall, these data support the potential prognostic value of ctDNA when combining with other risk factors/features.

## Discussion

The W&W strategy has been widely used in the treatment of LARC patients undergoing nCRT. However, it has been reported that the local recurrence was about 25% in patients adopting the “W&W” approach [31], in contrast with only 3% in patients receiving surgery and being confirmed to be pCR [5]. Notably, a recent study showed that in patients receiving the W&W approach, DM occurred in 36% of patients with local regrowth (LR) compared with only 1% in patients without LR (*P* < 0.001) [10]. Two possible mechanisms may explain the high DM incidence in LR patients: The regrowing cancer cells disseminate to distant organs, or LR is just a high-risk indicator of DM. For the first scenario, reducing LR should reduce the risk of DM. Commonly used tools for nCRT response assessment include DRE, MRI, and endoscopy. Given that 8% to 15% of patients with non-cCR were confirmed to be pCR [7,32], the accuracy of clinical assessment needs to be improved. On the other hand, the clinical assessment may also misclassify non-pCR as cCR, which increases the risk of LR. For example, Sclafani and colleagues found that mrTRG and pTRG had low concordance [33], and patients with mrTRG1 could be pTRG1 or pTRG2 (Dworak criteria [34], corresponding to TRG3 or TRG4 in our study). Similarly, another study showed that the agreement between MRI staging and pathological staging was only about 50%, and 14% of patients were understaging by MRI [35]. In our study, the accuracy of MRI for predicting pCR/non-pCR was 70.6% (84/119), and 10 cCR (mrTRG1) patients were indeed non-pCR, including 3 pTRG1, 5 pTRG2, and 2 pTRG3. These data indicate that even non-pCR patients with poor pTRG could be misclassified by MRI, highlighting the urgency of improving its predictive performance. Unlike the traditional local examination tool, ctDNA is a systemic biomarker and can detect micrometastasis or minimal residual disease (MRD) earlier than imaging [36–38]. By tracking ctDNA clearance, we showed that 50% (4/8, among 89 patients with ctDNA clearance data) of patients misclassified as cCR by MRI could be corrected by ctDNA non-clearance, which indicates the existence of MRD. Furthermore, when combining ctDNA and MRI information, the power to predict pCR/non-pCR was improved in which AUC increased from 0.73 (MRI only) to 0.89 (ctDNA combining MRI). These results suggest that ctDNA can supplement imaging tools to improve preoperative assessment. Unfortunately, although there should be MRD in non-pCR patients, due to decrease of tumor burden after nCRT, ctDNA could not be detected in certain proportion of non-pCR patients making it difficult to differentiate these non-pCR patients from pCR patients. As a result, future studies could try higher ctDNA sequencing depth and use of unique molecular identifier to increase the detection resolution and enhance signal–noise ratio to increase its ability to detect minimal residual tumor. Also, the value of ctDNA in the “W&W” strategy needs to be further validated in clinical trials to check whether adding ctDNA testing as one of the assessment tools can reduce the incidence of LR and further decrease the risk of DM and prolong patient’s overall survival in patients undergoing “W&W” approach.

ctDNA clearance may indicate the tumor burden rather than the malignant degree, while recurrence is determined by both of residual tumor burden and tumor growth speed [39]. As a result, despite its close relationship with pCR/non-pCR, ctDNA clearance itself seems not to be a sufficient biomarker to predict recurrence. Besides, there was only 1 time point and lack of serial ctDNA testing after surgery, which may limit the ability of ctDNA clearance in indicating recurrence, since for tumor recurrence, the increase of tumor burden is a gradual process, ctDNA clearance 5 to 12 days after surgery (Time5) does not mean patient will not relapse in the future. On the other hand, detection of driver mutations after treatment may indicate high tumor malignancy and could be a strong and independent risk factor for recurrence. Our results were in line with a study of Tie and colleagues [15], which showed that the detection of the mutations of 15 potential colorectal driver genes after nCRT or after surgery predicted a worse RFS. In fact, a total of 7 patients had detectable driver mutations at both Time4 (after nCRT) and Time5 (after surgery), and 6 out of 7 patients relapsed although receiving surgery and postoperative chemotherapy. Our results were consistent with another study of Tie and colleagues [24], suggesting that these high-risk patients might not benefit from standard treatments and more intensive treatments should be offered. Additionally, the combination of ctDNA and HR_feature could achieve better postoperative risk stratification for LARC patients undergoing nCRT and TME. Patients can be classified into low-risk, intermediate-risk, and high-risk groups according to the status of HR_feature and driver ctDNA detection. Customized intervention measures thereby can be applied on patients with various risk degrees.

There were several limitations in our study. Firstly, most patients were followed up for only 2 years, which might be insufficient for evaluating the prognosis of certain patients, especially for pCR patients. Secondly, the endoscopy and DRE information before surgery was lacking in our study, which may influence the complete evaluation of cCR. Thirdly, we did not monitor ctDNA dynamic changes during the follow-up period, so the advantage of ctDNA over traditional monitoring tools could not be evaluated. Fourthly, although it is so far the largest study focusing on ctDNA dynamic changes in LARC patients, the study lacked an independent validation cohort. Therefore, the results still need to be further validated by large high-quality prospective cohorts.

In summary, ctDNA combing with MRI information achieved better prediction for nCRT response and has a potential to help patient selection in “W&W” strategy. Large-scale clinical trial should be conducted to validate whether the addition of ctDNA testing in current clinical tools can improve long-term outcomes of patients undergoing “W&W” approach. Moreover, ctDNA combining with HR_ feature can also improve risk assessment for LARC patients undergoing nCRT.

## Supporting information

S1 REMARK checklist Remark checklist of the study. (DOCX)

S1 Analysis plan Analysis plan of the study. (DOCX)

S1 Text Supplementary methods. (DOCX)

S1 Table Gene list of the 422-gene panel. (DOCX)

S2 Table 15 potential colorectal cancer driver genes. (DOCX)

S3 Table Association of baseline clinicopathological features with the response to nCRT. (Table A) Univariable logistic regression of clinical features with pCR/non-pCR status (*n =* 119, non-pCR was designated as positive event). (Table B) Distribution of patients with certain clinical features in pCR and non-pCR groups (*n =* 119). Fisher exact test was used for significance test.
(DOCX)

S4 Table Association of ctDNA features with the response to nCRT. (Table A) Univariable logistic regression analysis of the genes detected to be mutated at baseline with pCR/non-pCR status (*n* = 119, non-pCR was designated as positive event). Only genes that were detected to be mutated in at least 6 patients at baseline were included in the analysis. (Table B) Univariable and multivariable logistic regression of the KEGG pathways detected to be mutated at baseline with pCR/non-pCR status (*n =* 119, non-pCR was designated as positive event). Only pathways that had at least 5 overlapping genes with detected mutated genes in the cohort and were mutated in at least 8 patients were included. There were 125 pathways to be included, and the table shows 6 pathways with *P* ≤ 0.05. (Table C) Genes that were detected to be mutated at baseline belong to HRR and HMT pathway. (Table D) Univariable logistic regression of acquired mutation status (*n* = 103) and T234_clearance (*n* = 89) with pCR/non-pCR status (non-pCR was designated as positive event). (Table E) Distribution of the patients with key features in various pTRG groups and pCR/non-pCR groups. Fisher exact test was used in the comparison between pCR and non-pCR groups, and Cochran–Armitage trend test was used for trend test.
(DOCX)

S5 Table Predictive model construction and calculation of risk score (n = 89). Models were constructed based on multivariable logistic regression. Features met selection criteria (statistical analysis in the “Materials and methods” section) were selected including age, *TP53* mutation status, HRR mutation status, HMT mutation status, acquired mutation status, T234_clearance status, and mrTRG grade. In multivariable logistic regression, age had >0.25 *P* value and was removed, and 6 features were finally selected for model construction. Three models were constructed and compared. Model 1 included ctDNA information only (5 features), model 2 included mrTRG information only (1 feature), and model 3 included both ctDNA and mrTRG information (6 features). Risk scores were calculated according to the coefficients of multivariable logistic regression. A total of 89 patients with detectable baseline gene mutations and serial ctDNA testing data (completed the whole study) were included in the analysis.
(DOCX)

S6 Table Postoperative recurrence risk analyzed by univariable and multivariable Cox regression (n = 89). A total of 89 patients with both baseline detectable gene mutations and serial ctDNA test data were included.
(DOCX)

S1 Fig Detectability at baseline was not associated with the response to nCRT and patient prognosis. nCRT, neoadjuvant chemoradiotherapy; pCR, pathological complete response; pTRG, pathological tumor regression grade.
(TIF)

S2 Fig Baseline mutation landscape. The landscape of high-frequency somatic genetic variations detected by ctDNA sequencing in the baseline plasma of the LARC patients (*n* = 119). Numbers in the left of the plot represent overall frequency as well as frequency in pCR and non-pCR groups [overall (pCR, non-pCR)]. ctDNA, circulating tumor DNA; HMT, histone methyltransferase; HRR, homologous recombination; LARC, locally advanced rectal cancer; pCR, pathological complete response; pTRG, pathological tumor regression grade.
(TIF)

S3 Fig (A) Distribution of ctDNA clearance in different clinicopathological groups (*n =* 89). For T234_clearance_status, “Clearance” means that the mutation with the highest VAF at baseline was disappeared (cleared) at all of Time2, Time3, and Time4 points, that is, was persistently cleared during nCRT. “Non-clearance” means that the mutation could be detected at least 1 time point. For clear display, “Non-clearance” was labeled by red color, and “Clearance” was labeled by gray color. The 8 arrows in the bottom of the plot indicate 8 patients who were classified to be cCR by MRI (mrTRG1) but were confirmed to be non-pCR after surgery. The 4 blue arrows indicate 4 of the above 8 patients who were ctDNA non-clearance, and the 4 yellow arrows indicate the other 4 patients who were ctDNA clearance. (B) The distribution of patients with acquired mutations in different clinicopathological groups (*n* = 103). cCR, clinical complete response; ctDNA, circulating tumor DNA; MRI, magnetic resonance imaging; mrTRG, magnetic resonance imaging tumor regression grade; nCRT, neoadjuvant chemoradiotherapy; pCR, pathological complete response; pTRG, pathological tumor regression grade; VAF, variant allele frequency.
(TIF)

S4 Fig Clearance of baseline HRR and HMT mutations during nCRT. Clearance of a mutation was defined as a baseline mutation was cleared at all of the 3 time points before surgery (Time2, Time3, and Time4). (A) Clearance of baseline HRR mutations during nCRT. (B) Clearance of baseline HMT mutations during nCRT. P×× represents patient ID (for example, P102). Mutations labeled by red color represent mutations that were not cleared. There were 1 HRR mutation and 1 HMT mutation, which were not cleared during nCRT. A total of 89 patients who had clearance data were included in the analysis. (C) Non-clearance rates of representative KEGG pathways. Only pathways with at least 15 mutations were included in the analysis. The plot shows top 5 pathways with the lowest non-clearance rate, top 5 pathways with the highest non-clearance rate, and top 5 pathways with most mutations. The red dash line represents overall non-clearance rate (11.9%). HRR, homologous recombination repair; HMT, histone methyltransferase family; KEGG, Kyoto Encyclopedia of Genes and Genomes; nCRT, neoadjuvant chemoradiotherapy.
(TIF)

S5 Fig Kaplan–Meier curves of the RFS based on detection of baseline *TP53* mutation (A) and *KRAS* mutation (B), 4 high-risk pathological features (C-F), PNI, tumor deposits, vascular invasion, and lymph node metastasis. HR, hazard ratio; *KRAS*_mut, *KRAS* mutation; *KRAS*_wt, *KRAS* wild type; PNI, perineural invasion; RFS, recurrence-free survival; *TP53*_mut, *TP53* mutation; *TP53*_wt, *TP53* wild type; 95% CI, 95% confidence interval.
(TIF)

S6 Fig (A) Kaplan–Meier curves of the RFS based on Time4 clearance status (T4 represents Time4); (B) Kaplan–Meier curves of the RFS based on Time5 clearance status stratified by HR_feature status (HR_feature (+) represents high-risk feature positive, T5 represents Time5). A total of 89 patients with detectable baseline mutations and serial ctDNA testing data were included in the analysis. ctDNA, circulating tumor DNA; HR, hazard ratio; HR_feature, high-risk feature; nCRT, neoadjuvant chemoradiotherapy; RFS, recurrence-free survival; 95% CI, 95% confidence interval.
(TIF)

## Abbreviations

AJCC: American Joint Committee on Cancer
AUC: area under the curve
cCR: clinical complete response
95% CI: 95% confidence interval
CRC: colorectal cancer
ctDNA: circulating tumor DNA
DM: distant metastasis
DRE: digital rectal examination
FDA: Food and Drug Administration
HMT: histone methyltransferase
HR: hazard ratio
HR_feature: high-risk feature
HRR: homologous recombination
KEGG: Kyoto Encyclopedia of Genes and Genomes
LARC: locally advanced rectal cancer
LR: local regrowth
MRI: magnetic resonance imaging
mrTRG: magnetic resonance imaging tumor regression grade
NCCN: National Comprehensive Cancer Network
nCRT: neoadjuvant chemoradiotherapy
PNI: perineural invasion
pCR: pathological complete response
pTRG: pathological tumor regression grade
RFS: recurrence-free survival
ROC: receiver operating characteristic
TME: total mesorectal excision
VAF: variant allele frequency
W&W: Watch and Wait
