# Supplementary material for: Utility of ctDNA in predicting response to neoadjuvant chemoradiotherapy and prognosis assessment in locally advanced rectal cancer: A prospective cohort study
Source: PLoS Med. 2021 Aug 31;18(8):e1003741. doi: 10.1371/journal.pmed.1003741 (PMC8407540; doi:10.1371/journal.pmed.1003741)
Supplement: S1 Analysis plan — (DOCX) [file pmed.1003741.s002.docx]

**Utility of ctDNA in Predicting Response to Neoadjuvant Chemoradiotherapy and Prognosis Assessment in Locally Advanced Rectal Cancer：A Prospective Cohort Study**

**Statistical analysis plan**

**Aim 1 –** to investigate the association of clinicopathological features and ctDNA features with the response to nCRT.

- Distribution of patients with certain baseline clinicopathological features in pCR/non-PCR group, features including age, sex, clinical stage, MRF, EVMI and mrTRG grade (Fisher exact test). Association of baseline clinicopathological features with the pCR/non-pCR status (logistic regression).
- Distribution of patients with certain baseline ctDNA features in pCR/non-pCR groups or various pTRG groups (Fisher exact test and Cochran-Armitage trend test). Association of baseline ctDNA features with pCR/non-pCR status (logistic regression). Baseline ctDNA features include detectability of baseline mutations, detection of gene mutation, and detection of pathway mutations.
- Distribution of dynamic ctDNA features in pCR/non-pCR groups and various pTRG groups (Fisher exact test and Cochran-Armitage trend test). Association of dynamic ctDNA features with pCR/non-pCR status (logistic regression). Dynamic ctDNA features included ctDNA clearance and status of acquired mutation during nCRT. ctDNA clearance was defined as undetection of the mutation with the highest VAF at baseline at all of the three time points before surgery (T234_clearance).

**Aim 2 -** to investigate whether ctDNA features alone or combined with MRI information can predict pCR/non-pCR status, or improve the predictive performance of MRI alone.

- According to the results of the analyses in aim 1, select baseline and ctDNA dynamic features which are significantly associated with pCR status to perform multivariable logistic regression.
- Based on the results of multivariable logistic regression, construct predictive models for pCR/non-pCR status. That is, to calculate linear predictor (risk score) for non-pCR to predict patients’ risk for non-pCR
- Construct three models which include ctDNA information only, mrTRG information only, and both of ctDNA information and mrTRG, and evaluate the performance of the three models by ROC method.
- Use internal cross validation to validate the three models

**Aim 3** – to investigate whether baseline and dynamic ctDNA features can predict patients’ recurrence after surgery

- Whether ctDNA clearance after nCRT and after surgery can predict patients’ recurrence-free survival (RFS) (Kaplan–Meier estimation and Cox regression model).
- Whether detection of ctDNA at baseline is associated with patients’ RFS (Kaplan–Meier estimation and Cox regression model).
- Whether detection of mutations of certain genes (TP53, APC, KRAS) is associated with patients’ RFS (Kaplan–Meier estimation and Cox regression model)

**Changes or additional analyses following results from initial analysis**

- According to the number of the patients who completed baseline ctDNA sequencing, following serial ctDNA sequencing, and had detectable baseline mutations, we adjusted included patients for each analysis. All analyses only involving baseline features included all of the 119 patients who completed baseline ctDNA sequencing, for example, association of baseline features with pCR status or RFS. All analyses only involving serial ctDNA test data included 103 patients who completed serial ctDNA sequencing, for example, association of acquired mutation with pCR status. All analyses involving ctDNA clearance (no matter other features were involved) included 89 patients who completed baseline and serial ctDNA sequencing, at the same time, had detectable mutations at baseline, because ctDNA clearance was defined as the clearance of the mutation with the highest VAF at baseline during nCRT. If a patient had no detectable mutations at baseline, ctDNA clearance tracking was unavailable in this patient.
- After feature selection, 6 features were selected for predictive model construction including baseline TP53 mutation status, baseline HRR (homologous recombination repair) mutation status, baseline HMT (histone methyltransferase) mutation status, T234_clearance, acquired mutation status and mrTRG. Model with ctDNA information only included the first 5 features; model with mrTRG information only included the mrTRG feature, and the combining model included all of the 6 features.
- A newly published study showed that detection of 15 potential colorectal cancer gene mutations after nCRT or surgery was associated with a worse RFS (Tie J, et al. Gut. 2019;68(4):663-71. doi: 10.1136/gutjnl-2017-315852). We thereby investigated whether detection of 15 potential colorectal cancer driver genes can predict patients’ RFS in our cohort (Kaplan–Meier estimation and Cox regression model).
- After initial survival analysis, it was found ctDNA clearance or detection of potential CRC driver gene mutations can only explain part of the patients’ recurrence, a certain proportion of patients with recurrence were negative in the two features. To seek the underlying cause of the recurrence in these patients, we further investigated how postoperative pathological characteristics affected patients’ recurrence including lymph node metastasis, tumor deposit, perineural invasion and vascular invasion, and found majority of patients with recurrence were positive in one or more of the above pathological features; we thereby defined a “high-risk feature”, to represent positive in at least one of the above pathological features as well as baseline TP53 and KRAS mutation. By combining ctDNA features with high-risk feature, recurrence prediction and risk stratification were further improved.

**Additional analyses through review process**

- Feature selection process was further systematically clarified and associated figures and tables were provided in revised manuscript.
- Clearance of HRR and HMT mutation during nCRT was analysed and compared with other KEGG pathways.
