# Supplementary material for: Utility of ctDNA in predicting response to neoadjuvant chemoradiotherapy and prognosis assessment in locally advanced rectal cancer: A prospective cohort study
Source: PLoS Med. 2021 Aug 31;18(8):e1003741. doi: 10.1371/journal.pmed.1003741 (PMC8407540; doi:10.1371/journal.pmed.1003741)
Supplement: S1 Text — (DOCX) [file pmed.1003741.s003.docx]

#### S1 Text. Supplementary Methods

#### Patients and sample collection

#### A total of 119 patients with LARC (cT3-4, N0-2, M0) who were treated in the Department of Radiation Oncology, Fudan University Shanghai Cancer Center were enrolled in the study.

**1.1 Inclusion criteria**

• Pathologically confirmed adenocarcinoma of rectum.

• staged as T3-4 and/or N+ at baseline.

• Distance from anus ≤ 12cm.

• No evidence of distant metastasis at baseline.

• Age 18-75 years; male or female

• ECOG performance status 0-1.

• UGT1A1*28 genotype is 6/6 or 6/7.

• No chemotherapy or any other immune-therapy was used before enrollment.

• Subject is willing and able to comply with the protocol for the duration of the study including undergoing treatment, blood sample collection, scheduled visits and examinations including follow up.

• Willing and able to provide written informed consent/assent for the study.

**1.2. Exclusion criteria**

• Pregnant or breast-feeding women.

• Patients with prior malignancies in the past 5 years, except for the skin basal cell carcinoma and cervical carcinoma in situ.

• Patients with uncontrolled seizures or psychiatric disorders.

• Patients with severe or active heart diseases, such as symptomatic coronary heart disease, congestive cardiac failure (NYHA ≥ II), severe arrhythmia or myocardial infarction in the past 12 months.

• Patients who received organ transplantation or are treated with immunosuppressive therapy.

• Patients with severe uncontrolled infection or other concomitant diseases.

• Baseline routine blood test biochemical indexes not meeting the following criteria: Hemoglobin (Hb) ≥ 90g/L, Absolute neutrophil count (ANC) ≥ 1.5×10^9^/L, Platelet (PLT) ≥ 100×10^9^/L, Aspartate aminotransferase (AST), Alanine aminotransferase (ALT), Alkaline phosphatase (ALP) ≤ 2.5×upper limit of normal (ULN), Total bilirubin (TBIL) ≤ 1.5×ULN, Creatinine (Cr) ≤ 1×ULN, Albumin (ALB) ≥ 30 g/L.

• Dihydropyrimidine dehydrogenase (DPD) -deficient patients.

• UGT1A1*28 genotype is 7/7.

• Patients who are allergic to any component of drugs in this study.

#### 1.3. Treatment

#### The patients received capecitabine/irinotecan radiosensitized nCRT regimen. The detailed information of this regimen was as follows.

**1.3.1. Neoadjuvant chemoradiation (nCRT) stage**

Radiotherapy: whole pelvic IMRT DT 50Gy/25Fx

Concurrent chemotherapy:

Capecitabine: 625 mg/m^2^ bid D1-5 qw

Irinotecan: 80 mg/m^2^ D1 qw (UGT1A1*28 6/6) or 65 mg/m^2^ D1 qw (UGT1A1*28 6/7)

**1.3.2. Interval chemotherapy stage**

Two weeks after the completion of nCRT, a cycle of interval chemotherapy (CAPIRI) is performed.

CAPIRI: Capecitabine 1000 mg/m^2^ bid D1-14 + Irinotecan 200 mg/m^2^ D1

**1.3.3. Operative stage**

Surgical treatment is performed about 6-8 weeks after the nCRT. The specific surgical method is decided by surgeons.

**1.3.4. Adjuvant chemotherapy stage**

A total of five courses of adjuvant chemotherapy (CAPOX) are performed.

CAPOX: Capecitabine 1000 mg/m^2^ bid D1-14 q3w + Oxaliplatin 130 mg/m^2^ D1 q3w

#### This regimen was from a phase III randomized controlled clinical trial (CinClare, NCT02605265), which proved that by capecitabine + irinotecan regimen, the patients achieved a pCR rate of 30%, in contrast with a pCR rate of 15% in the control arm in which only capecitabine was used. And the adverse effects were acceptable and under controlled. The most common grade 3-4 toxicities were leukopenia, neutropenia, and diarrhea. These toxicities could be controlled if the patients with adverse effects were treated in time. The clinical outcome report of the CinClare trial has recently been published in Journal of Clinical Oncology [1]. Although capecitabine/irinotecan regimen is still not routinely recommended in guidelines, it has entered the clinical practice in our cancer center.

#### 1.4. Sample collection

#### Plasma samples were collected at the following time points: before nCRT (Time1), at the 15^th^fraction of nCRT (Time2), at the 25^th^fraction of nCRT (Time3), 0-1d before surgery (Time4) and 5-12 d after surgery (Time5). For each sample, 8-10ml of peripheral (intravenous) blood was collected and stored in a 10ml BD EDTA-K2 anticoagulation tube. Plasma was isolated within 2 hours by centrifugation (1800g, 10 minutes). The collected plasma was removed into a 1.5ml low-adsorption centrifuge tube (Eppendorf DNA Lobind tube). The tube was sealed and stored at -80℃ for future use. Sample collection schedule was shown in Figure1.

#### Baseline plasma samples were collected in all 119 patients. But 16 patients couldn’t fulfill the following 4 time-points of sample collection and 103 patients completed the whole course. Finally, a total of 531 plasma samples and 119 matched leukocyte germline control samples were collected and applied to NGS panel sequencing. Of the 103 completed patients, 89 patients with detectable baseline mutations were analyzed for mutation clearance analysis, whereas 103 patients were examined for acquire mutation analysis.

1. **DNA Extraction, hybridization capture and sequencing**

cfDNA was extracted using the NucleoSpin Plasma XS kit (Macherey Nagel) with optimized manufacturer’s protocols. Whole blood DNAs were extracted using the DNeasy Blood & Tissue kit (Qiagen) according to the manufacturer’s protocols. Purified DNA was qualified by Nanodrop2000 (Thermo Fisher Scientific) and quantified by Qubit 2.0 using the dsDNA HS Assay Kit (Life Technologies) according to the manufacturer’s recommendations. Sequencing libraries were prepared using the KAPA Hyper Prep kit (KAPA Biosystems) according to the manufacturer’s protocol.

A customized NGS panel targeting 422 cancer-related genes was used for hybridization enrichment. In brief, indexed DNA libraries were pooled together to a total amount of 2 μg and subjected to probe-based hybridization using IDT xGen Lockdown reagents (IDT, Coralville, IA) and Dynabeads M-270 (Thermo Fisher). Captured libraries were on-beads amplified with Illumina p5 and p7 primers in KAPA HiFiHotStartReadyMix (KAPA Biosystems). The final library was quantified by KAPA Library Quantification kit (KAPA Biosystems) per manufacturer’s instructions. Bioanalyzer 2100 (Agilent, Stanta Clara, CA) was used to determine the fragment size distribution of the final library. The target-enriched library was then sequenced on Illumina HiSeq4000 NGS platforms (Illumina) according to the manufacturer’s instructions. The average sequencing depth of blood control and cfDNA samples was ~200X and ~4000X, respectively.

1. **Sequence data processing, mutation and bioinformatics analysis**

Trimmomatic[2] was used for FASTQ file quality control (QC). Leading/trailing low quality (quality reading below 30) or N bases were removed. Reads from each sample were mapped to the reference sequence hg19 (Human Genome version 19) using Burrows-Wheeler Aligner (BWA-mem, v0.7.12) [3] with parameters (-t 8 -M). Local realignment around indels and base quality score recalibration was applied with the Genome Analysis Toolkit (GATK 3.4.0)[4].

Samtools[5] was employed for detection of candidate somatic mutations in cfDNA samples. For cfDNA samples, we required minimum variant allele frequency= 0.5%, minimum variant supporting reads = 5. We required somatic p-value = 0.1, minimum quality score = 30 and variant supporting reads mapped to both strands with strand bias no greater than 10%. The resulted mutation lists were further filtered through an internally collected list (1000 normal samples) of recurrent artifacts on the same sequencing platform. Specifically, if a variant was detected (≥5 mutant reads) in >10% of the standard normal samples, it was considered a likely systematic artifact and was removed. Mutations were also removed if they were present in >1% population frequency in the 1000 Genomes Project or 65000 exomes project (ExAC). The pipeline (0.5% AF detection limit) was validated to achieved specificity > 99.99% using a 20 cfDNA samples from healthy persons.

References

1. Zhu J, Liu A, Sun X, Liu L, Zhu Y, Zhang T, et al. Multicenter, Randomized, Phase III Trial of Neoadjuvant Chemoradiation With Capecitabine and Irinotecan Guided by UGT1A1 Status in Patients With Locally Advanced Rectal Cancer. J Clin Oncol. 2020;38(36):4231-9. doi: 10.1200/JCO.20.01932. PubMed PMID: 33119477; PubMed Central PMCID: PMCPMC7768334.

2. Bolger AM, Lohse M, Usadel B. Trimmomatic: a flexible trimmer for Illumina sequence data. Bioinformatics. 2014;30(15):2114-20. doi: 10.1093/bioinformatics/btu170. PubMed PMID: 24695404; PubMed Central PMCID: PMC4103590.

3. Li H, Durbin R. Fast and accurate short read alignment with Burrows-Wheeler transform. Bioinformatics. 2009;25(14):1754-60. doi: 10.1093/bioinformatics/btp324. PubMed PMID: 19451168; PubMed Central PMCID: PMC2705234.

4. Van der Auwera GA, Carneiro MO, Hartl C, Poplin R, Del Angel G, Levy-Moonshine A, et al. From FastQ data to high confidence variant calls: the Genome Analysis Toolkit best practices pipeline. Current protocols in bioinformatics. 2013;43:11 0 1- 0 33. doi: 10.1002/0471250953.bi1110s43. PubMed PMID: 25431634; PubMed Central PMCID: PMC4243306.

5. Li H, Handsaker B, Wysoker A, Fennell T, Ruan J, Homer N, et al. The Sequence Alignment/Map format and SAMtools. Bioinformatics. 2009;25(16):2078-9. doi: 10.1093/bioinformatics/btp352. PubMed PMID: 19505943; PubMed Central PMCID: PMC2723002.
