# Supplementary material for: Utility of ctDNA in predicting response to neoadjuvant chemoradiotherapy and prognosis assessment in locally advanced rectal cancer: A prospective cohort study
Source: PLoS Med. 2021 Aug 31;18(8):e1003741. doi: 10.1371/journal.pmed.1003741 (PMC8407540; doi:10.1371/journal.pmed.1003741)
Supplement: S1 Table — (DOCX) [file pmed.1003741.s004.docx]

**S1 Table. 422 gene panel**

| ABCB1 (MDR1) | CASP8 | DAXX | FLCN | KIF1B | NAT2 | PPP2R1A | SGK1 | TUBB2A |
| --- | --- | --- | --- | --- | --- | --- | --- | --- |
| ABCB4 | CBL | DDR2 | FLT1 (VEGFR1) | KIF5B | NBN | PRDM1 | SLC34A2 | TUBB2B |
| ABCC2 (MRP2) | CBLB | DENND1A | FLT3 | KIT | NCOR1 | PRF1 | SLC7A8 | TUBB3 |
| ADH1A | CC2D2B | DHFR | FLT4 | KITLG | NF1 | PRKACA | SMAD2 | TUBB4A |
| ADH1B | CCND1 | DHFRL1 | FOXA1 | KLLN | NF2 | PRKACG | SMAD3 | TUBB4B |
| ADH1C | CCNE1 | DICER1 | FOXP1 | KMT2A (MLL) | NFE2L2 | PRKAR1A | SMAD4 | TUBB6 |
| AIP | CD274 (PD-L1) | DNMT3A | FRG1 | KMT2B | NFKBIA | PRKCI | SMAD7 | TYMS |
| AKT1 | CD74 | DPYD | GATA1 | KMT2C | NKX2-1 | PRKDC | SMARCA4 | U2AF1 |
| AKT2 | CDA | DUSP2 | GATA2 | KMT2D (MLL2) | NKX2-2 | PRSS1 | SMARCB1 | UGT1A1 |
| AKT3 | CDC73 | EGFR | GATA3 | KRAS | NKX2-4 | PRSS3 | SMO | VEGFA |
| ALDH2 | CDH1 | EML4 | GATA4 | LHCGR | NOTCH1 | PTCH1 | SOS1 | VHL |
| ALK | CDK10 | EP300 | GATA6 | LMO1 | NOTCH2 | PTEN | SOX1 | WAS |
| AMER1 | CDK12 | EPAS1 | GNA11 | LRP1B | NOTCH3 | PTK2 | SOX14 | WISP3 |
| APC | CDK4 | EPCAM | GNA15 | LYN | NPM1 | PTPN11 | SOX2 | WRN |
| AR | CDK6 | EPHA2 | GNAQ | LZTR1 | NQO1 | PTPN13 | SOX21 | WT1 |
| ARAF | CDK8 | EPHA3 | GNAS | MAP2K1 (MEK1) | NRAS | PTPRD | SOX3 | XPA |
| ARID1A | CDKN1A | EPHA5 | GRIN2A | MAP2K2 (MEK2) | NRG1 | QKI | SPOP | XPC |
| ARID1B | CDKN1B | EPHB2 | GRM3 | MAP2K4 | NSD1 | RAC1 | SPRY4 | XRCC1 |
| ARID2 | CDKN1C | ERBB2(HER2) | GRM8 | MAP3K1 | NTRK1 | RAC3 | SRC | YAP1 |
| ARID5B | CDKN2A | ERBB2IP | GSTM1 | MAP3K4 | NTRK3 | RAD50 | SRY | ZNF2 |
| ASCL4 | CDKN2B | ERBB3 | GSTM4 | MAP4K3 | PAK3 | RAD51 | STAG2 | ZNF217 |
| ASXL1 | CDKN2C | ERBB4 | GSTM5 | MAX | PALB2 | RAD51C | STAT3 | ZNF703 |
| ATF1 | CEBPA | ERCC1 | GSTP1 | MCL1 | PALLD | RAD51D | STK11 |  |
| ATIC | CEBPB | ERCC2 | GSTT1 | MDM2 | PARK2 | RAF1 | STMN1 |  |
| ATM | CEBPD | ERCC3 | HDAC2 | MDM4 | PARP1 | RARA | STT3A |  |
| ATR | CEP57 | ERCC4 | HDAC9 | MECOM | PARP2 | RARG | SUFU |  |
| ATRX | CHD4 | ERCC5 | HGF | MED12 | PAX5 | RASGEF1A | TEK |  |
| AURKA | CHEK1 | ESR1 | HLA-A | MEF2B | PBRM1 | RB1 | TEKT4 |  |
| AURKB | CHEK2 | ETV1 | HNF1A | MEN1 | PDCD1 (PD1) | RECQL4 | TERC |  |
| AXIN2 | CLEC2D | ETV4 | HNF1B | MET | PDCD1LG2 (PD-L2) | RELN | TERT |  |
| AXL | CREBBP | EWSR1 | HRAS | MGMT | PDE11A | RET | TET2 |  |
| BAI3 | CRKL | EXT1 | HSD3B1 | MITF | PDGFRA | RHOA | TGFBR2 |  |
| BAK1 | CSF1R | EXT2 | IDH1 | MLH1 | PDGFRB | RICTOR | THADA |  |
| BAP1 | CTCF | EZH2 | IDH2 | MLH3 | PDK1 | RNF43 | TMEM127 |  |
| BARD1 | CTLA4 | FANCA | IGF1R | MLLT1 | PGR | ROS1 | TMPRSS2 |  |
| BCL2 | CTNNB1 | FANCC | IGF2 | MLLT3 | PHOX2B | RPTOR | TNFAIP3 |  |
| BCL2L11(BIM) | CUL3 | FANCD2 | IKBKE | MLLT4 | PIK3C3 | RRM1 | TNFRSF11A |  |
| BCR | CUX1 | FANCE | IKZF1 | MPL | PIK3CA | RUNX1 | TNFRSF14 |  |
| BIRC3 | CXCR4 | FANCF | IL7R | MRE11A | PIK3R1 | RUNX1T1 | TNFRSF19 |  |
| BLM | CYLD | FANCG | INPP4B | MSH2 | PIK3R2 | RUNX3 | TNFSF11 |  |
| BMPR1A | CYP19A1 | FANCL | IRF2 | MSH6 | PKHD1 | SBDS | TOP1 |  |
| BRAF | CYP2A13 | FANCM | JAK1 | MTHFR | PLAG1 | SDC4 | TOP2A |  |
| BRCA1 | CYP2A6 | FAT1 | JAK2 | MTOR | PLK1 | SDHA | TP53 |  |
| BRCA2 | CYP2A7 | FBXW7 | JAK3 | MUTYH | PMS1 | SDHB | TP63 |  |
| BRD4 | CYP2B6*6 | FGF19 | JARID2 | MYC | PMS2 | SDHC | TPMT |  |
| BRIP1 | CYP2C19*2 | FGFR1 | JUN | MYCL | POLD1 | SDHD | TSC1 |  |
| BTG2 | CYP2C9*3 | FGFR2 | KDM5A | MYCN | POLD3 | SEPT9 | TSC2 |  |
| BTK | CYP2D6 | FGFR3 | KDM6A | MYD88 | POLE | SETBP1 | TSHR |  |
| BUB1B | CYP3A4*4 | FGFR4 | KDR (VEGFR2) | MYH9 | POLH | SETD2 | TTF1 |  |
| c11orf30 | CYP3A5 | FH | KEAP1 | NAT1 | POT1 | SF3B1 | TUBB |  |
