# Supplementary material for: Utility of ctDNA in predicting response to neoadjuvant chemoradiotherapy and prognosis assessment in locally advanced rectal cancer: A prospective cohort study
Source: PLoS Med. 2021 Aug 31;18(8):e1003741. doi: 10.1371/journal.pmed.1003741 (PMC8407540; doi:10.1371/journal.pmed.1003741)
Supplement: S2 Table — (DOCX) [file pmed.1003741.s005.docx]

**S2 Table. 15 driver genes of colorectal cancer**

| SMAD4 |
| --- |
| TP53 |
| AKT1 |
| APC |
| BRAF |
| CTNNB1 |
| ERBB3 |
| FBXW7 |
| HRAS |
| KRAS |
| NRAS |
| PIK3CA |
| PPP2R1A |
| RNF43 |
| POLE |
