# Supplementary material for: Utility of ctDNA in predicting response to neoadjuvant chemoradiotherapy and prognosis assessment in locally advanced rectal cancer: A prospective cohort study
Source: PLoS Med. 2021 Aug 31;18(8):e1003741. doi: 10.1371/journal.pmed.1003741 (PMC8407540; doi:10.1371/journal.pmed.1003741)
Supplement: S3 Table — (Table A) Univariable logistic regression of clinical features with pCR/non-pCR status (n = 119, non-pCR was designated as positive event). (Table B) Distribution of patients with certain clinical features in pCR and non-pCR groups (n = 119). Fisher exact test was used for significance test. (DOCX) [file pmed.1003741.s006.docx]

**S3 Table A. Univariable logistic regression of clinical features with pCR/non-pCR status (n=119)**

| **Feature** | **Feature items** | **Coefficient** | **Odds ratio (95%CI)** | **P value** |
| --- | --- | --- | --- | --- |
| Sex | Female |  | 1 (reference) |  |
|  | Male | 0.01 | 1.05(0.45-2.4) | 0.90 |
| *Age |  | 0.05 | 1.05(1.01-1.09) | 0.02 |
| MRF | Negative |  | 1 (reference) |  |
|  | Positive | 0.07 | 1.07(0.45-2.64) | 0.88 |
| EVMI | Negative |  | 1 (reference) |  |
|  | Positive | 0.19 | 1.21(0.54-2.81) | 0.65 |
| Clinical stage | Stage IIA-IIIB |  | 1 (reference) |  |
|  | Stage IIIC | 0.50 | 1.64(0.72-3.98) | 0.25 |
| *mrTRG^1^ |  | 1.17 | 3.24(1.83-6.21) | <0.001 |

Note: non-pCR was designated as positive event. * Features with *P*≤ 0.1;

pCR: pathological complete response; mrTRG: magnetic resonance imaging tumor regression grade; MRF: mesorectal fascia. EVMI: extramural vascular invasion.

^1^mrTRG was modeled as a continuous variable.

**S3 Table B. Distribution of patients with certain clinical features in pCR and Non-pCR group (n=119)**

| **Features** | **Feature items** | **Non-pCR** | **pCR** | **Total** | **P value** |
| --- | --- | --- | --- | --- | --- |
| **Clinicopathological features** | | | | | |
| Age (median) |  | 58.5 | 54 | 57 | 0.035 |
| Sex | Female | 22(0.65,0.28) | 12(0.35,0.29) | 34(1,0.29) | 1 |
|  | Male | 56(0.66,0.72) | 29(0.34,0.71) | 85(1,0.71) |  |
|  | Total | 78(0.66,1) | 41(0.34,1) | 119(1,1) |  |
| Clinical stage | IIA | 1(0.5,0.01) | 1(0.5,0.02) | 2(1,0.02) | 0.60 |
|  | IIIA | 1(1,0.01) | 0(0,0) | 1(1,0.01) |  |
|  | IIIB | 49(0.62,0.63) | 30(0.38,0.73) | 79(1,0.66) |  |
|  | IIIC | 27(0.73,0.35) | 10(0.27,0.24) | 37(1,0.31) |  |
|  | Total | 78(0.66,1) | 41(0.34,1) | 119(1,1) |  |
| EVMI* | Negative | 52(0.64,0.67) | 29(0.36,0.71) | 81(1,0.68) | 0.69 |
|  | Positive | 26(0.68,0.33) | 12(0.32,0.29) | 38(1,0.32) |  |
|  | Total | 78(0.66,1) | 41(0.34,1) | 119(1,1) |  |
| MRF* | Negative | 58(0.65,0.74) | 31(0.35,0.76) | 89(1,0.75) | 1 |
|  | Positive | 20(0.67,0.26) | 10(0.33,0.24) | 30(1,0.25) |  |
|  | Total | 78(0.66,1) | 41(0.34,1) | 119(1,1) |  |

pCR: pathological complete response; MRF: mesorectal fascia. EVMI: extramural vascular invasion.
