# Supplementary material for: Utility of ctDNA in predicting response to neoadjuvant chemoradiotherapy and prognosis assessment in locally advanced rectal cancer: A prospective cohort study
Source: PLoS Med. 2021 Aug 31;18(8):e1003741. doi: 10.1371/journal.pmed.1003741 (PMC8407540; doi:10.1371/journal.pmed.1003741)
Supplement: S4 Table — (Table A) Univariable logistic regression analysis of the genes detected to be mutated at baseline with pCR/non-pCR status (n = 119, non-pCR was designated as positive event). Only genes that were detected to be mutated in at least 6 patients at baseline were included in the analysis. (Table B) Univariable and multivariable logistic regression of the KEGG pathways detected to be mutated at baseline with pCR/non-pCR status (n = 119, non-pCR was designated as positive event). Only pathways that had at least 5 overlapping genes with detected mutated genes in the cohort and were mutated in at least 8 patients were included. There were 125 pathways to be included, and the table shows 6 pathways with P ≤ 0.05. (Table C) Genes that were detected to be mutated at baseline belong to HRR and HMT pathway. (Table D) Univariable logistic regression of acquired mutation status (n = 103) and T234_clearance (n = 89) with pCR/non-pCR status (non-pCR was designated as positive event). (Table E) Distribution of the patients with key features in various pTRG groups and pCR/non-pCR groups. Fisher exact test was used in the comparison between pCR and non-pCR groups, and Cochran–Armitage trend test was used for trend test. (DOCX) [file pmed.1003741.s007.docx]

**S4 Table A. Univariable logistic regression analysis of the genes detected to be mutated at baseline with pCR/non-pCR status (n=119)**

| **Feature** | **Coefficient** | **Odds ratio** | **P value** | **BH-adjusted P value** |
| --- | --- | --- | --- | --- |
| *POLD1 | -1.66 | 0.19(0.03-0.92) | 0.05 | >0.25 |
| *APC | 0.91 | 2.48(0.91-7.98) | 0.09 |  |
| *TP53 | 1.10 | 3.02(0.92-13.63) | 0.10 |  |
| NSD1 | -1.41 | 0.24(0.03-1.31) | 0.11 |  |
| KMT2B | -0.99 | 0.37(0.07-1.76) | 0.21 |  |
| NF1 | 1.01 | 2.74(0.42-53.46) | 0.37 |  |
| GRIN2A | -0.68 | 0.51(0.09-2.85) | 0.42 |  |
| NOTCH1 | 0.29 | 1.34(0.27-9.62) | 0.74 |  |
| KRAS | 0.19 | 1.21(0.37-4.69) | 0.77 |  |
| GNAS | 0.05 | 1.05(0.2-7.84) | 0.95 |  |

Note: non-pCR was designated as positive event. * Genes with *P*≤ 0.1. Only genes which were detected to be mutated in at least 6 patients at baseline were included in the analysis. pCR: pathological complete response. BH-adjusted: Benjamini-Hochberg-adjusted

**S4 Table B. Univariable and multivariable logistic regression of the KEGG pathways detected to be mutated at baseline with pCR/non-pCR status (n=119)**

| **Univariable logistic regression analysis** | | | | | **Multivariable logistic regression analysis** | | |
| --- | --- | --- | --- | --- | --- | --- | --- |
| **Pathway** | **Coefficient** | **Odds ratio** | **P value** | **BH-adjusted P value** | **Coefficient** | **Odds ratio** | **P value** |
| *Homologous recombination repair (HRR) | -1.35 | 0.26(0.08-0.76) | 0.02 | >0.25 | -1.35 | 0.26(0.07-0.85) | 0.03 |
| *Histone methyltransferases (HMT) (or lysine degradation) | -1.19 | 0.31(0.1-0.87) | 0.03 |  | -1.26 | 0.28(0.09-0.89) | 0.03 |
| Adherens junction | -1.04 | 0.35(0.14-0.88) | 0.03 |  | -0.94 | 0.39(0.12-1.29) | 0.12 |
| Osteoclast differentiation | -1.87 | 0.15(0.02-0.7) | 0.03 |  | -1.89 | 0.15(0.01-1.46) | 0.13 |
| Influenza A | -1.26 | 0.28(0.08-0.91) | 0.04 |  | 0.39 | 1.48(0.22-10.85) | 0.69 |
| Th17 cell differentiation | -1.46 | 0.23(0.05-0.94) | 0.05 |  | -0.46 | 0.63(0.1-3.91) | 0.62 |

Note: non-pCR was designated as positive event. * Pathways with *P*≤ 0.05. Only pathways which had at least 5 overlapping genes with detected mutated genes in the cohort and were mutated in at least 8 patients were included. There were 125 pathways to be included and the table shows 6 pathways with *P*≤0.05. pCR: pathological complete response; BH-adjusted: Benjamini-Hochberg-adjusted; KEGG: Kyoto Encyclopedia of Genes and Genomes.

**S4 Table C. Genes which were detected to be mutated at baseline belong to HRR and HMT pathway**

| **Pathway or protein family** | **Genes mutated in the cohort** |
| --- | --- |
| Homologous recombination repair (HRR) | ATM, BRIP1, POLD1, NBN, BRCA1, BRCA2, RAD50, BLM, RAD51C, PALB2, RAD51 |
| Histone methyltransferase family (HMT) | KMT2A, KMT2C, KMT2B, EZH2, SETD2, NSD1 |

**S4 Table D. Univariable logistic regression of acquired mutation status (n=103) and T234_clearance (n=89)**

| **Feature** | **Coefficient** | **Odds ratio** | **P value** |
| --- | --- | --- | --- |
| *Acquired mutation | 1.71 | 5.56(1.03-103.31) | 0.10 |
| *T234_clearance | -2.19 | 0.11(0.01-0.6) | 0.04 |

Note: non-pCR was designated as positive event. * Features with *P*≤ 0.1

**S4 Table E. Distribution of the patients with or without key features in various pTRG groups and pCR/non-pCR group**

| **Distribution in various pTRG groups (Cochran Armitage trend test)** | | | | | | | |
| --- | --- | --- | --- | --- | --- | --- | --- |
|  | pTRG0 (n=41) | pTRG1 (n=12) | pTRG2 (n=53) | pTRG3 (n=13) | Total (n=119) | Trend test | P value |
| APC mutation | 5 (12.2%) | 2 (16.7%) | 15 (28.3%) | 3 (23.1%) | 25 (21.0%) | Increasing | 0.04 |
| TP53 mutation | 3 (7.3%) | 2 (16.7%) | 10 (18.9%) | 3 (23.1%) | 18 (15.1%) | Increasing | 0.04 |
| KRAS mutation | 4 (9.8%) | 2 (16.7%) | 5 (9.4%) | 2 (15.4%) | 13 (10.9%) | Increasing | 0.41 |
| POLD1 mutation | 5 (12.2%) | 0 (0.0%) | 2 (3.8%) | 0 (0.0%) | 7 (5.9%) | Decreasing | 0.03 |
| HRR mutation | 10 (24.4%) | 2 (16.7%) | 4 (7.5%) | 0 (0.0%) | 16 (13.4%) | Decreasing | 0.002 |
| HMT mutation | 10 (24.4%) | 1 (8.3%) | 3 (5.7%) | 3 (23.1%) | 17 (14.3%) | Decreasing | 0.06 |
|  |  |  |  |  |  |  |  |
|  | pTRG0 (n=26) | pTRG1 (n=12) | pTRG2 (n=52) | pTRG3 (n=13) | Total (n=103) | Trend test | P value |
| Acquired mutation | 1 (3.8%) | 1 (8.3%) | 10 (19.2%) | 3 (23.1%) | 15 (14.6%) | Increasing | 0.02 |
|  |  |  |  |  |  |  |  |
|  | pTRG0 (n=23) | pTRG1 (n=9) | pTRG2 (n=45) | pTRG3 (n=12) | Total (n=89) | Trend test | P value |
| T234_clearance | 22 (95.7%) | 7 (77.8%) | 32 (71.1%) | 8 (66.7%) | 69 (77.5%) | Decreasing | 0.008 |
|  |  |  |  |  |  |  |  |
| **Distribution in pCR and non-pCR group (Fisher exact test)** | | | | | | | |
|  | pCR (n=41) | non-pCR (n=78) | Total (n=119) | P value |  | | |
| APC mutation | 5 (12.2%) | 20 (25.6%) | 25 (21.0%) | 0.1 |  |  |  |
| TP53 mutation | 3 (7.3%) | 15 (19.2%) | 18 (15.1%) | 0.11 |  |  |  |
| KRAS mutation | 4 (9.8%) | 9 (11.5%) | 13 (10.9%) | 1 |  |  |  |
| POLD1 mutation | 5 (12.2%) | 2 (2.6%) | 7 (5.9%) | 0.05 |  |  |  |
| HRR mutation | 10 (24.4%) | 6 (7.7%) | 16 (13.4%) | 0.02 |  |  |  |
| HMT mutation | 10 (24.4%) | 7 (9.0%) | 17 (14.3%) | 0.03 |  |  |  |
|  |  |  |  |  |  |  |  |
|  | pCR (n=26) | non-pCR (n=77) | Total (n=103) | P value |  |  |  |
| Acquired mutation | 1 (3.8%) | 14 (18.2%) | 15 (14.6%) | 0.11 |  |  |  |
|  |  |  |  |  |  |  |  |
|  | pCR (n=23) | non-pCR (n=66) | Total (n=89) | P value |  |  |  |
| T234_clearance | 22 (95.7%) | 47 (71.2%) | 69 (77.5%) | 0.02 |  |  |  |

pTRG: pathological tumor regression grade; pCR: pathological complete response; HRR: Homologous recombination repair; HMT: Histone methyltransferase family
