# Supplementary material for: Utility of ctDNA in predicting response to neoadjuvant chemoradiotherapy and prognosis assessment in locally advanced rectal cancer: A prospective cohort study
Source: PLoS Med. 2021 Aug 31;18(8):e1003741. doi: 10.1371/journal.pmed.1003741 (PMC8407540; doi:10.1371/journal.pmed.1003741)
Supplement: S5 Table — Models were constructed based on multivariable logistic regression. Features met selection criteria (statistical analysis in the “Materials and methods” section) were selected including age, TP53 mutation status, HRR mutation status, HMT mutation status, acquired mutation status, T234_clearance status, and mrTRG grade. In multivariable logistic regression, age had >0.25 P value and was removed, and 6 features were finally selected for model construction. Three models were constructed and compared. Model 1 included ctDNA information only (5 features), model 2 included mrTRG information only (1 feature), and model 3 included both ctDNA and mrTRG information (6 features). Risk scores were calculated according to the coefficients of multivariable logistic regression. A total of 89 patients with detectable baseline gene mutations and serial ctDNA testing data (completed the whole study) were included in the analysis. (DOCX) [file pmed.1003741.s008.docx]

**S5 Table. Predictive model construction and calculation of risk score (n=89)**

| **Model 1: ctDNA information only (5 features)** | | | |
| --- | --- | --- | --- |
| **Feature** | **Coefficient** | **Odds ratio** | **P value** |
| TP53 mutation status | 1.62 | 5.04(0.95-47.96) | 0.09 |
| HRR mutation status | -1.69 | 0.18(0.04-0.75) | 0.02 |
| HMT mutation status | -1.91 | 0.15(0.03-0.62) | 0.01 |
| Acquired mutation status | 1.61 | 5.01(0.7-105.21) | 0.17 |
| T234_clearance status | -2.28 | 0.1(0.01-0.64) | 0.04 |
| Risk score = 3.31 (intercept)+1.62×TP53 mutation status + (-1.69)×HRR mutation status + (-1.91)×HMT mutation status +1.61×acquire mutation status + (-2.28)×T234_clearance status | | | |

ctDNA: circulating tumor DNA; HRR: Homologous recombination repair; HMT: Histone methyltransferase family

| **Model 2: MRI information only (1 features)** | | | |
| --- | --- | --- | --- |
| **Feature** | **Coefficient** | **Odds ratio** | **P value** |
| mrTRG grade | 1.41 | 4.1(1.90-10.40) | 0.001 |
| Risk score = -1.76 (intercept)+1.41×mrTRG grade | | | |

mrTRG: magnetic resonance imaging tumor regression grade

| **Model 3: ctDNA+MRI (6 features)** | | | |
| --- | --- | --- | --- |
| **Feature** | **Coefficient** | **Odds ratio** | **P value** |
| TP53 mutation status | 1.41 | 4.09(0.69-42.46) | 0.16 |
| HRR mutation status | -1.56 | 0.21(0.04-1.04) | 0.06 |
| HMT mutation status | -1.75 | 0.17(0.03-0.83) | 0.04 |
| Acquired mutation status | 1.59 | 4.88(0.65-104.82) | 0.18 |
| T234_clearance status | -2.71 | 0.07(0-0.52) | 0.03 |
| mrTRG grade | 1.48 | 4.4(1.78-14.19) | 0.004 |
| Risk score = 0.67 (intercept)+1.41×TP53 mutation status + (-1.56)×HRR mutation status + (-1.75)×HMT mutation status +1.59×acquired mutation status + (-2.71)×T234_clearance status + 1.48×mrTRG grade | | | |

Non-pCR was designated as positive event. ctDNA: circulating tumor DNA; MRI: magnetic resonance imaging; HRR: homologous recombination repair; HMT: histone methyltransferase family; mrTRG: magnetic resonance imaging tumor regression grade
