# Supplementary material for: Utility of ctDNA in predicting response to neoadjuvant chemoradiotherapy and prognosis assessment in locally advanced rectal cancer: A prospective cohort study
Source: PLoS Med. 2021 Aug 31;18(8):e1003741. doi: 10.1371/journal.pmed.1003741 (PMC8407540; doi:10.1371/journal.pmed.1003741)
Supplement: S6 Table — A total of 89 patients with both baseline detectable gene mutations and serial ctDNA test data were included. (DOCX) [file pmed.1003741.s009.docx]

**S6 Table. Postoperative recurrence risk analyzed by univariable and multivariable Cox regression (n=89)**

| **Univariable Cox regression** | | | | **Multivariable Cox regression** | | |
| --- | --- | --- | --- | --- | --- | --- |
| **Feature** | **Hazard ratio** | **95%CI** | **P value** | **Hazard ratio** | **95%CI** | **P value** |
| Group (pCR vs. non-pCR) | 0.086 | 0.012--0.645 | 0.017 | 0.496 | 0.062--3.992 | 0.51 |
| HR_feature (Yes vs.No) | 7.248 | 2.133--24.63 | 0.002 | 12.73 | 1.635--99.07 | 0.015 |
| Time5_ctDNA_clearance | 0.434 | 0.141--1.332 | 0.145 | 0.535 | 0.166--1.723 | 0.294 |
| Time5_driver_mutation_detection | 14.329 | 5.351--38.37 | <0.001 | 11.94 | 3.697--38.57 | <0.001 |

pCR: pathological complete response; ctDNA: circulating tumor DNA; HR_feature: high-risk feature; 95%CI: 95% confidence interval.
