# Supplementary figures and images for: Utility of ctDNA in predicting response to neoadjuvant chemoradiotherapy and prognosis assessment in locally advanced rectal cancer: A prospective cohort study
Source: PLoS Med. 2021 Aug 31;18(8):e1003741. doi: 10.1371/journal.pmed.1003741 (PMC8407540; doi:10.1371/journal.pmed.1003741)

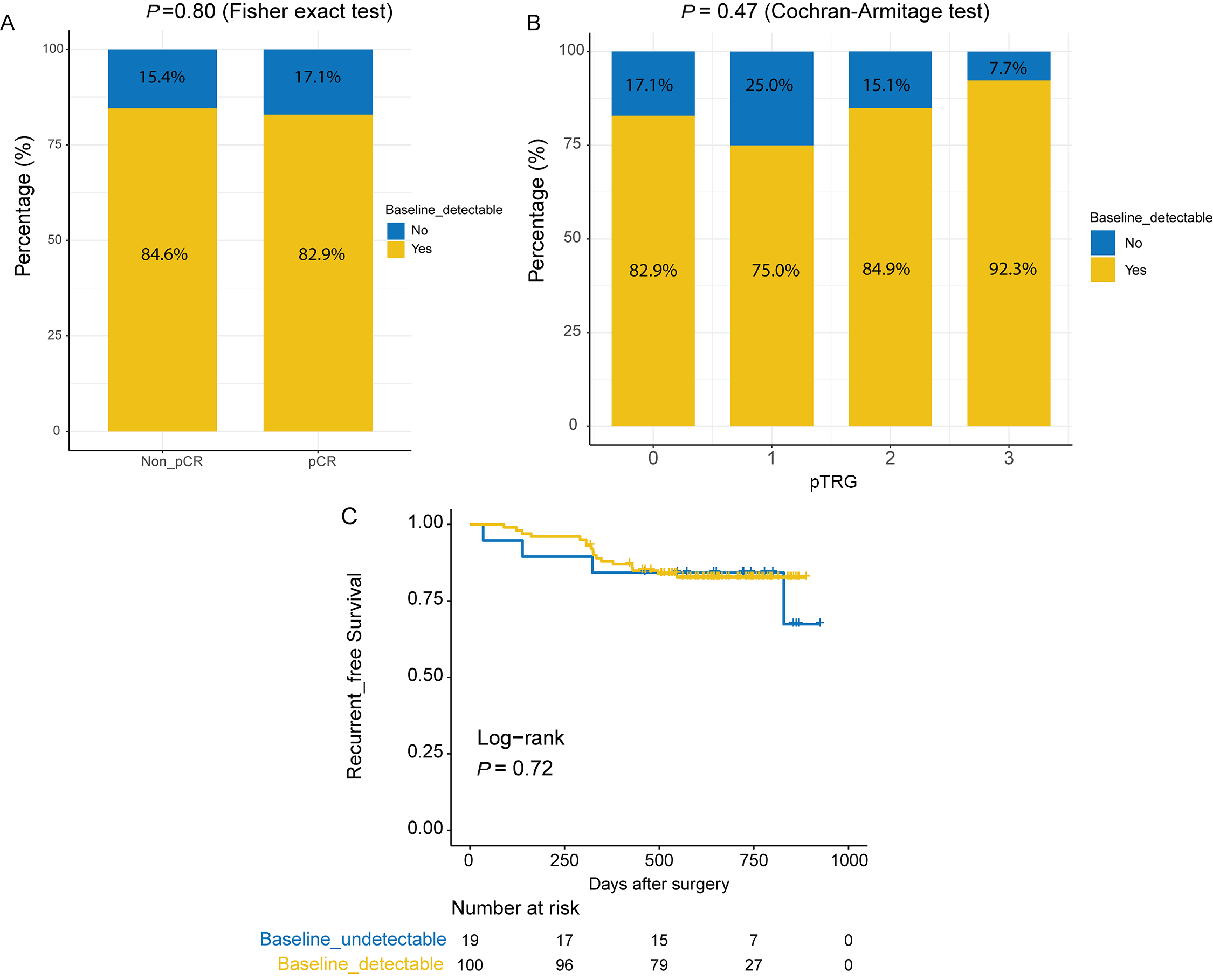

Supplement: S1 Fig — nCRT, neoadjuvant chemoradiotherapy; pCR, pathological complete response; pTRG, pathological tumor regression grade. (TIF) [file pmed.1003741.s010.tif]

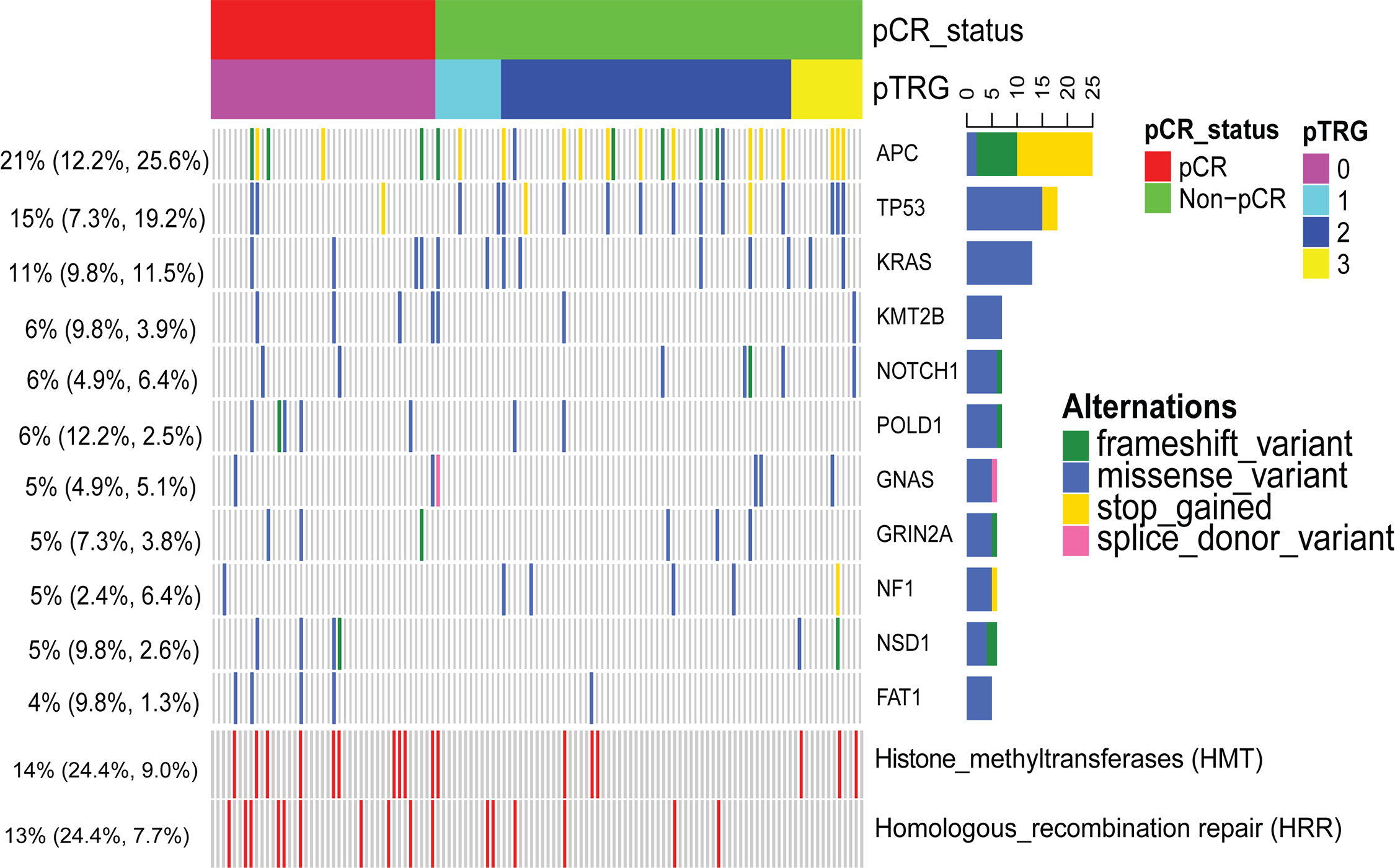

Supplement: S2 Fig — The landscape of high-frequency somatic genetic variations detected by ctDNA sequencing in the baseline plasma of the LARC patients (n = 119). Numbers in the left of the plot represent overall frequency as well as frequency in pCR and non-pCR groups [overall (pCR, non-pCR)]. ctDNA, circulating tumor DNA; HMT, histone methyltransferase; HRR, homologous recombination; LARC, locally advanced rectal cancer; pCR, pathological complete response; pTRG, pathological tumor regression grade. (TIF) [file pmed.1003741.s011.tif]

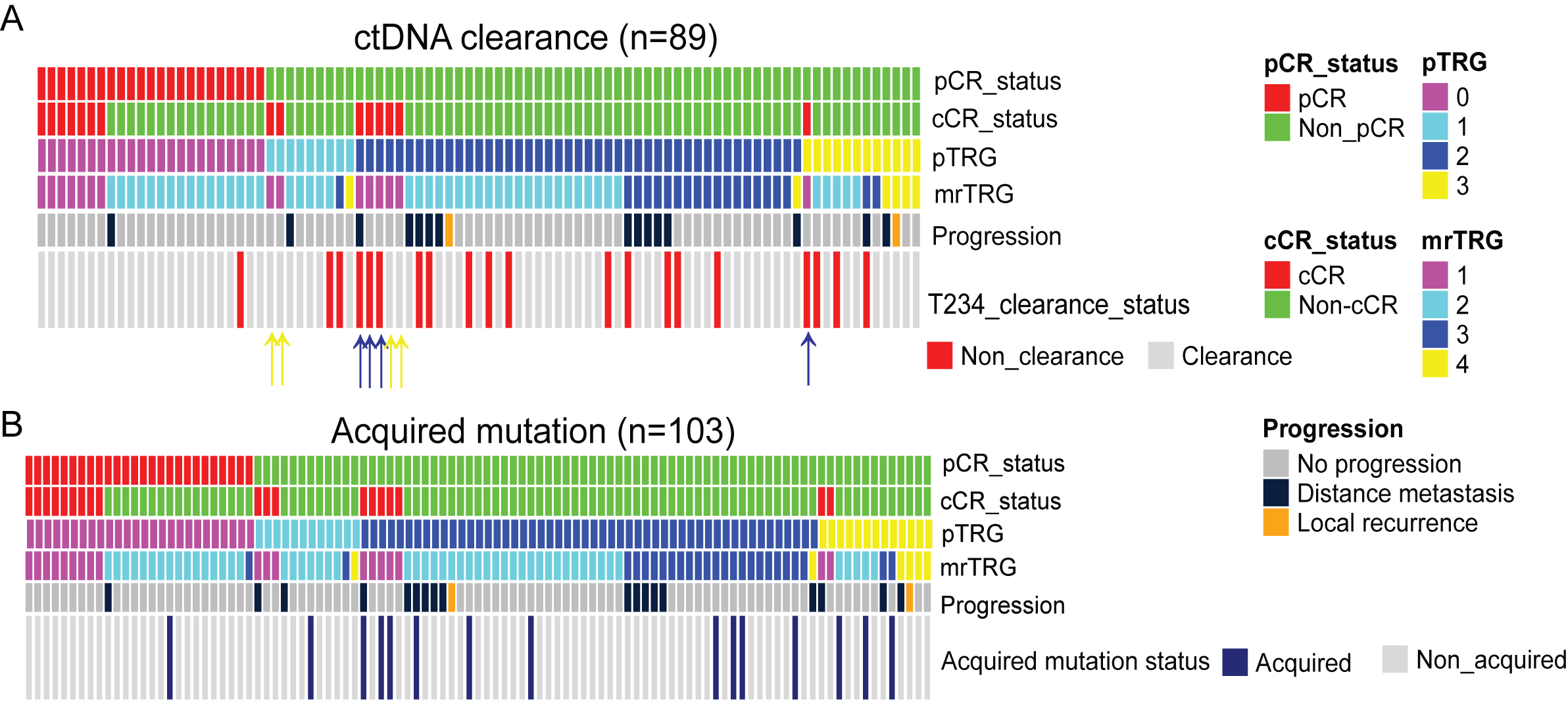

Supplement: S3 Fig — (A) Distribution of ctDNA clearance in different clinicopathological groups (n = 89). For T234_clearance_status, “Clearance” means that the mutation with the highest VAF at baseline was disappeared (cleared) at all of Time2, Time3, and Time4 points, that is, was persistently cleared during nCRT. “Non-clearance” means that the mutation could be detected at least 1 time point. For clear display, “Non-clearance” was labeled by red color, and “Clearance” was labeled by gray color. The 8 arrows in the bottom of the plot indicate 8 patients who were classified to be cCR by MRI (mrTRG1) but were confirmed to be non-pCR after surgery. The 4 blue arrows indicate 4 of the above 8 patients who were ctDNA non-clearance, and the 4 yellow arrows indicate the other 4 patients who were ctDNA clearance. (B) The distribution of patients with acquired mutations in different clinicopathological groups (n = 103). cCR, clinical complete response; ctDNA, circulating tumor DNA; MRI, magnetic resonance imaging; mrTRG, magnetic resonance imaging tumor regression grade; nCRT, neoadjuvant chemoradiotherapy; pCR, pathological complete response; pTRG, pathological tumor regression grade; VAF, variant allele frequency. (TIF) [file pmed.1003741.s012.tif]

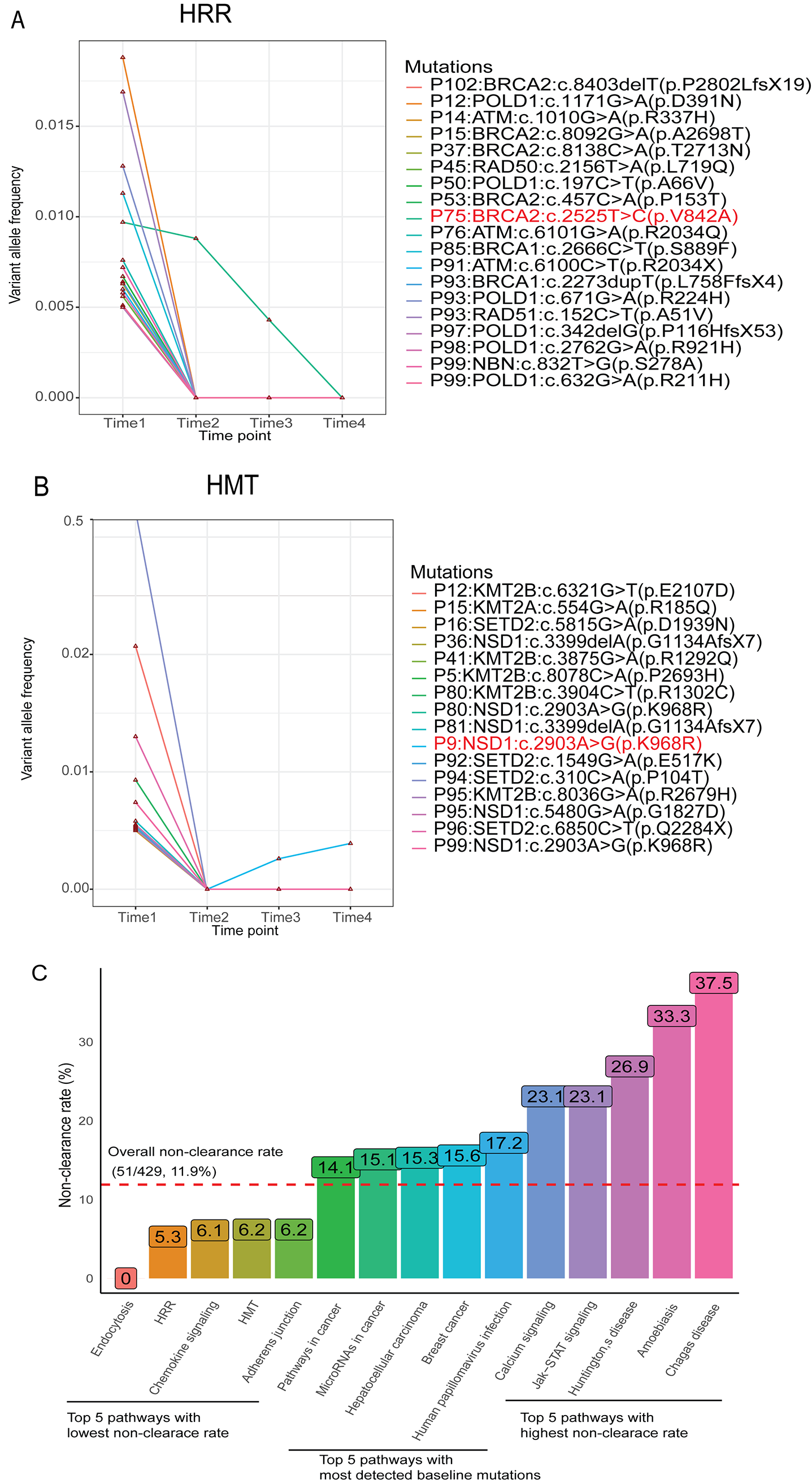

Supplement: S4 Fig — Clearance of a mutation was defined as a baseline mutation was cleared at all of the 3 time points before surgery (Time2, Time3, and Time4). (A) Clearance of baseline HRR mutations during nCRT. (B) Clearance of baseline HMT mutations during nCRT. P×× represents patient ID (for example, P102). Mutations labeled by red color represent mutations that were not cleared. There were 1 HRR mutation and 1 HMT mutation, which were not cleared during nCRT. A total of 89 patients who had clearance data were included in the analysis. (C) Non-clearance rates of representative KEGG pathways. Only pathways with at least 15 mutations were included in the analysis. The plot shows top 5 pathways with the lowest non-clearance rate, top 5 pathways with the highest non-clearance rate, and top 5 pathways with most mutations. The red dash line represents overall non-clearance rate (11.9%). HRR, homologous recombination repair; HMT, histone methyltransferase family; KEGG, Kyoto Encyclopedia of Genes and Genomes; nCRT, neoadjuvant chemoradiotherapy. (TIF) [file pmed.1003741.s013.tif]

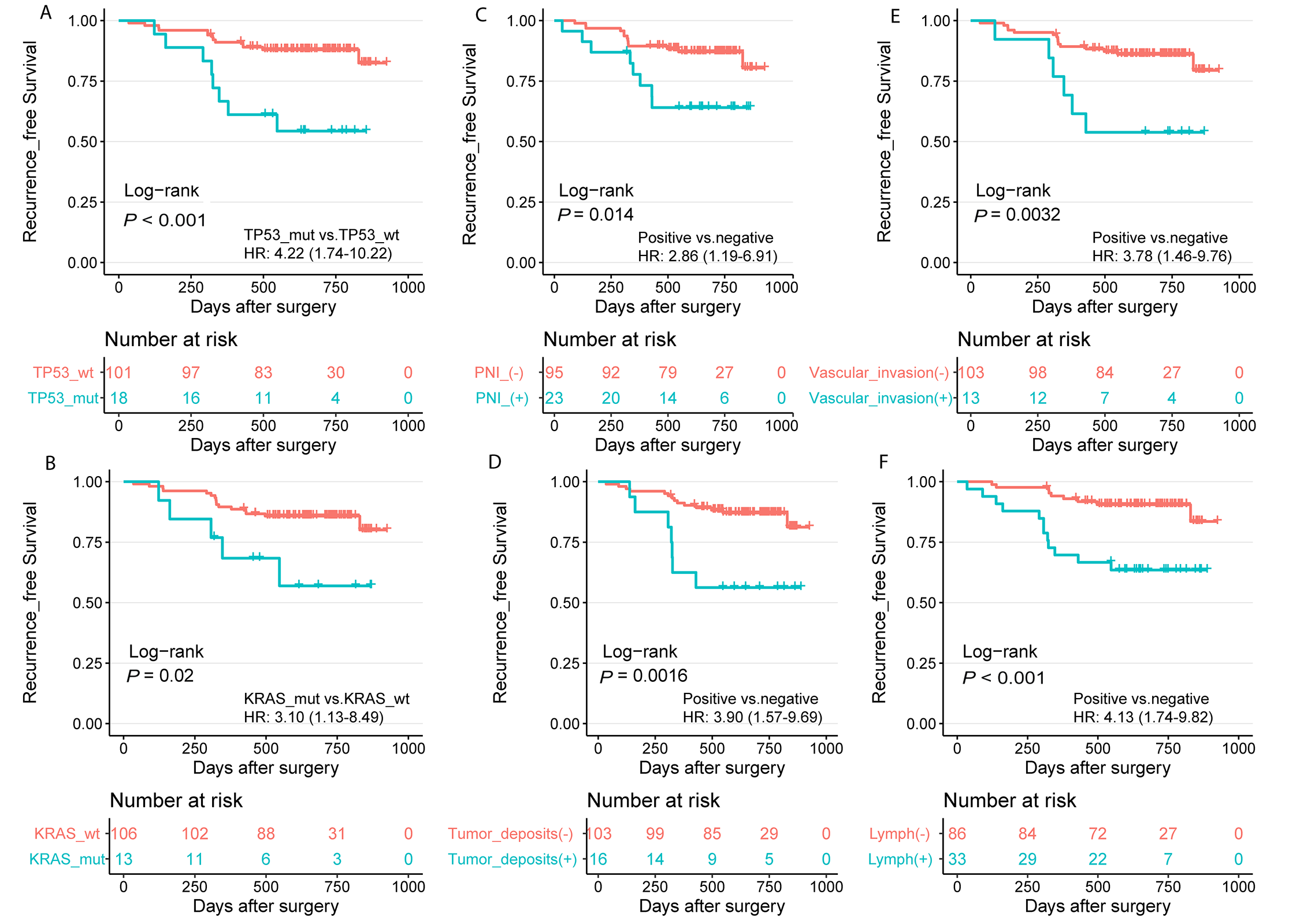

Supplement: S5 Fig — Kaplan–Meier curves of the RFS based on detection of baseline TP53 mutation (A) and KRAS mutation (B), 4 high-risk pathological features (C-F), PNI, tumor deposits, vascular invasion, and lymph node metastasis. HR, hazard ratio; KRAS_mut, KRAS mutation; KRAS_wt, KRAS wild type; PNI, perineural invasion; RFS, recurrence-free survival; TP53_mut, TP53 mutation; TP53_wt, TP53 wild type; 95% CI, 95% confidence interval. (TIF) [file pmed.1003741.s014.tif]

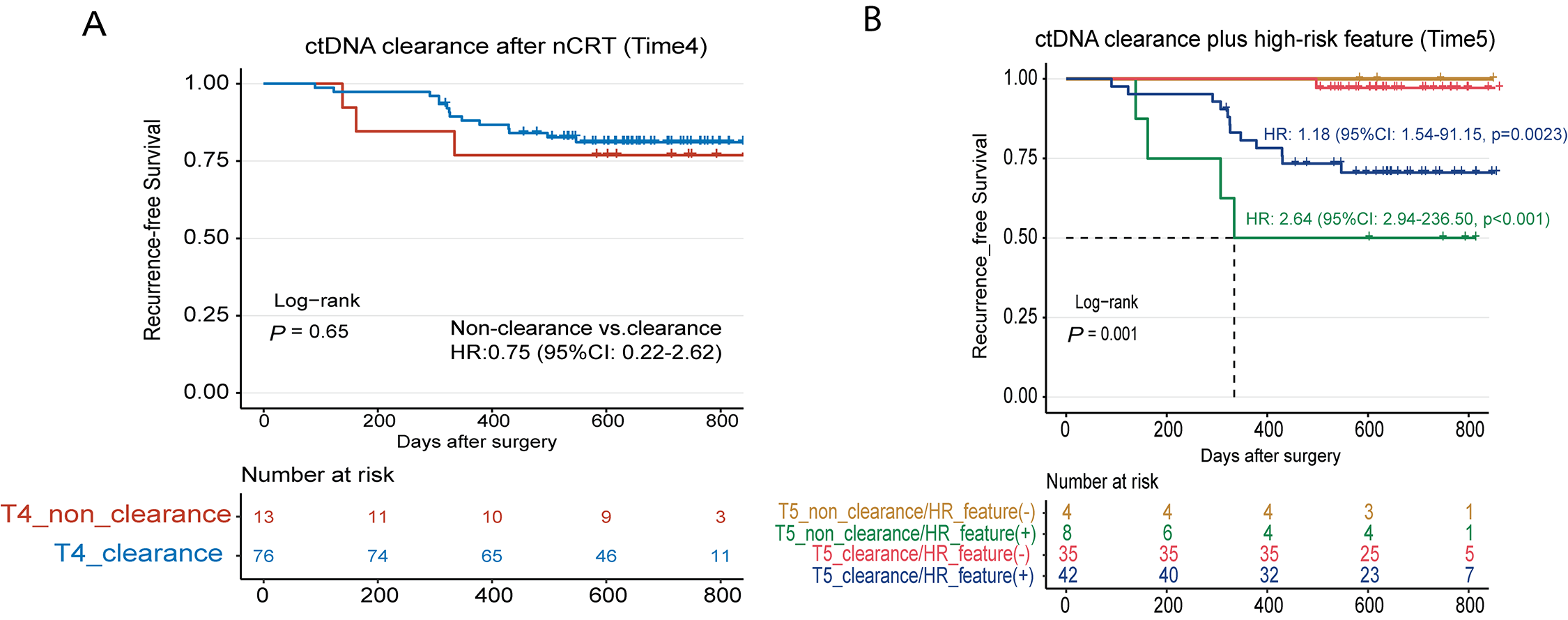

Supplement: S6 Fig — (A) Kaplan–Meier curves of the RFS based on Time4 clearance status (T4 represents Time4); (B) Kaplan–Meier curves of the RFS based on Time5 clearance status stratified by HR_feature status (HR_feature (+) represents high-risk feature positive, T5 represents Time5). A total of 89 patients with detectable baseline mutations and serial ctDNA testing data were included in the analysis. ctDNA, circulating tumor DNA; HR, hazard ratio; HR_feature, high-risk feature; nCRT, neoadjuvant chemoradiotherapy; RFS, recurrence-free survival; 95% CI, 95% confidence interval. (TIF) [file pmed.1003741.s015.tif]
